# Evolutionary Diversity of Dus2 Enzymes Reveals Novel Structural and Functional Features among Members of the RNA Dihydrouridine Synthases Family

**DOI:** 10.3390/biom12121760

**Published:** 2022-11-26

**Authors:** Murielle Lombard, Colbie J. Reed, Ludovic Pecqueur, Bruno Faivre, Sabrine Toubdji, Claudia Sudol, Damien Brégeon, Valérie de Crécy-Lagard, Djemel Hamdane

**Affiliations:** 1 Laboratoire de Chimie des Processus Biologiques, CNRS-UMR 8229, Collège de France, Université Pierre et Marie Curie, 11 Place Marcelin Berthelot, CEDEX 05, 75231 Paris, France; 2 Department of Microbiology and Cell Science, University of Florida, Gainesville, FL 32611, USA; 3 IBPS, Biology of Aging and Adaptation, Sorbonne Université 7 quai Saint Bernard, CEDEX 05, 75252 Paris, France; 4 Genetics Institute, University of Florida, Gainesville, FL 32610, USA

**Keywords:** dihydrouridine, tRNA, dihydrouridine synthase, tRNA binding, phylogeny, AlphaFold, structural-protein-evolution

## Abstract

Dihydrouridine (D) is an abundant modified base found in the tRNAs of most living organisms and was recently detected in eukaryotic mRNAs. This base confers significant conformational plasticity to RNA molecules. The dihydrouridine biosynthetic reaction is catalyzed by a large family of flavoenzymes, the dihydrouridine synthases (Dus). So far, only bacterial Dus enzymes and their complexes with tRNAs have been structurally characterized. Understanding the structure-function relationships of eukaryotic Dus proteins has been hampered by the paucity of structural data. Here, we combined extensive phylogenetic analysis with high-precision 3D molecular modeling of more than 30 Dus2 enzymes selected along the tree of life to determine the evolutionary molecular basis of D biosynthesis by these enzymes. Dus2 is the eukaryotic enzyme responsible for the synthesis of D20 in tRNAs and is involved in some human cancers and in the detoxification of β-amyloid peptides in Alzheimer’s disease. In addition to the domains forming the canonical structure of all Dus, i.e., the catalytic TIM-barrel domain and the helical domain, both participating in RNA recognition in the bacterial Dus, a majority of Dus2 proteins harbor extensions at both ends. While these are mainly unstructured extensions on the N-terminal side, the C-terminal side extensions can adopt well-defined structures such as helices and beta-sheets or even form additional domains such as zinc finger domains. 3D models of Dus2/tRNA complexes were also generated. This study suggests that eukaryotic Dus2 proteins may have an advantage in tRNA recognition over their bacterial counterparts due to their modularity.

## 1. Introduction

**Figure 1:**
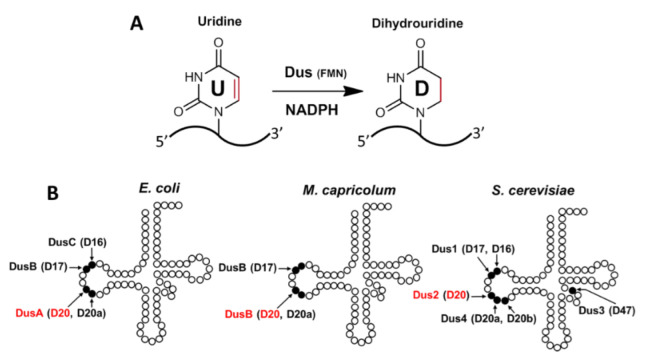
Dihydrouridine biosynthesis and localization of D residues in the tRNA. (**A**) Reduction reaction of uridine to dihydrouridine in tRNAs and mRNAs catalyzed by dihydrouridine synthases (Dus). These proteins are flavoenzymes that use FMN as a redox coenzyme and NADPH as a reductant source. (**B**) Cloverleaf secondary structure of the tRNAs shows the location of the D residues as well as the Dus enzymes that introduce them into *Escherichia coli*, *Mycoplasma capricolum*, and *Saccharomyces cerevisiae*.

Dihydrouridine (D) is one of the most abundant post-transcriptional modified bases in the transcriptome [1,2,3]. Present mainly in transfer RNA (tRNA) and occasionally in bacterial ribosomal RNA (rRNA), D has recently entered the messenger RNA (mRNA) world [1,2]. Indeed, this modification was recently detected in fission yeast mRNAs, including those encoding cytoskeleton-related proteins (2), in *Saccharomyces cerevisiae* mRNAs [4], but also in human mRNAs [5,6]. D is formed by the reduction of the C5 = C6 double bond of uridine, resulting in a loss of aromaticity, a unique feature among base modifications (Figure 1A). The lack of aromaticity leads to a pyrimidine that is unable to participate in stacking interactions [7,8]. 

The exact physiological role of D remains to be clearly defined, although some studies suggest that it plays an important role in 3D RNA shaping by promoting local flexibility and RNA backbone dynamics [9,10]. Consistent with this structural property, D is found primarily in single-stranded loops and in regions of the RNA that require flexibility, such as the tRNA elbow region, formed by the interaction between the D and TΨC loops and involving important tertiary interactions necessary to maintain its particular L-shaped structure [7,8]. Indeed, tRNAs lacking D in combination with other modifications have been shown to undergo rapid degradation [11], probably due to a defect in conformational flexibility. This could possibly explain how some cancer cells can prevent tRNA turnover by significantly increasing their D level in tRNAs [12] and thus promote cell growth [13]. In mRNAs, the absence of D has been shown to strongly affect meiotic chromosome segregation, leading to low gamete viability in yeast (2). In humans, D plays a role in the efficiency of the translation via a mechanism of action that remains to be established [5]. A relatively high level of D was observed in the tRNAs of cancer cells [12] and in those of psychrophilic organisms, where a greater demand for molecular flexibility is required [14].

D is often present at multiple positions in bacterial and eukaryotic tRNAs, and its abundance varies with both organism and tRNA type. In prokaryotes, D can be present at five positions of the tRNA (Figure 1B), namely positions 16, 17, 20 and 20a, all located in the D loop, but also at position 47 in the variable loop (V loop) [2], which has so far only been observed in *Bacillus subtilis* tRNA^Met^(CAU). In eukaryotes, D is observed in as many as six sites, including five in the D-loop (D16, D17, D20, D20a, and D20b) and one in the variable loop (D47) [3] (Figure 1B), with D20 being the most frequent D-modification in tRNAs [2]. D residues are introduced in tRNAs and mRNAs by a set of conserved dihydrouridine synthases (Dus) that are members of the Cluster of Orthologous Group family COG0042 [2,15,16,17]. These flavoenzymes catalyze an NADPH-dependent reduction of specific uridines using the flavin mononucleotide (FMN) as a tRNA-reducing coenzyme (Figure 1A) [2,15,16,18,19]. A phylogenetic study classified these enzymes into eight subfamilies, namely DusA, DusB, DusC, Dus1, Dus2, Dus3, Dus4, and archaeal Dus [20]. The first three enzymes are bacterial proteins, while Dus1 through Dus4 are found in eukaryotes, the last one being the unique member of Dus observed in archaea. Since DusB is present in almost all bacteria, a model where DusB is the bacterial ancestor, and DusA and DusC are the products of DusB duplication events that occurred shortly after the divergence of the major Proteobacteria groups was proposed [20]. In eukaryotes, Dus3 is considered the ancestral enzyme from which the other three are derived, starting with Dus2, followed by Dus1, and finally Dus4.

The tRNA-substrate specificity of Dus enzymes has been fully established in several model organisms, including *Mycoplasma capricolum* [21], *Escherichia coli* [15,19], and *Thermus thermophilus* [22,23] for prokaryotes, and *S. cerevisiae* [16,24], *S. pombe* [6], and humans [5,6,25] for eukaryotes. These studies revealed that Dus can generally modify up to three positions in a given tRNA substrate (Figure 1B). DusA, Dus1, and Dus4 are dual-site enzymes catalyzing the formation of D20/D20a, D16/D17, and D20a/D20b, respectively. In contrast, DusC, Dus2, and Dus3 can modify only one position and synthesize D17, D16, D20, and D47, respectively. Concerning DusB, we have recently shown that this enzyme can be either mono-site specific, such as the *E. coli* enzyme that catalyzes the synthesis of D17 [19], or tri-site specific, catalyzing D17, D20, and D20a in *M. capricolum* [21]. From an evolutionary standpoint, the mono-site specificity of a subset of Dus proteins could thus be regarded as a functional feature that has evolved recently, at least in the prokaryotes.

To date, three-dimensional structures, obtained by X-ray crystallography, are available in the PDB for *T. thermophilus* DusA [22], *E. coli* DusB [19], and *E. coli* DusC [26,27] (Table 1). The crystallographic structures of T. *thermophilus* DusA in complex with tRNA^Phe^ and *E. coli* DusC in complex with tRNA^Phe^ or tRNA^Trp^ were also solved [22,27]. These key data elucidated the structural and molecular basis of dihydrouridine biosynthesis in bacteria. In contrast, structural studies are limited for eukaryotic enzymes. The only data available to date are the structures of isolated domains of human Dus2 (hDus2 or Dus2L) [25,28,29] (Table 1). Beyond its physiological role, hDus2 seems to play a role in some cancers [13,30] but also in Alzheimer’s disease [31]. This enzyme promotes cell growth through its ability to interact with other enzymes, notably the aminoacyl tRNA synthetase complex EPRS [13] and the protein kinase R [30], by a mechanism that remains to be established. In addition, overexpression of hDus2 in tumorigenic cells appears to be associated with a poor prognosis for lung cancer patients [13].

**Figure 2:**
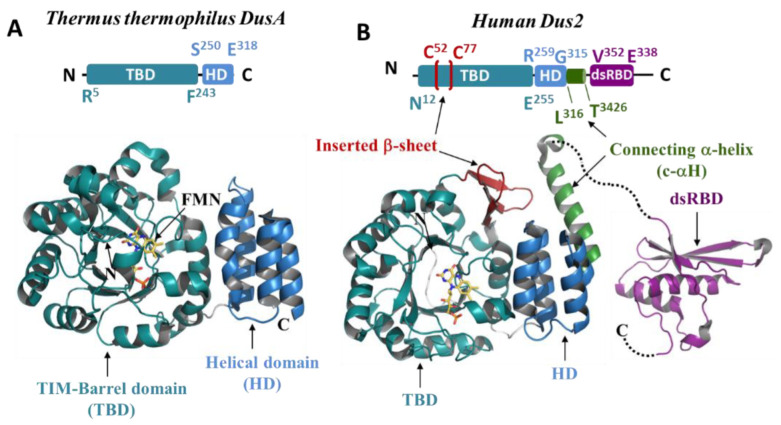
Crystallographic structures of Dus. (**A**,**B**) crystal structures of *T. thermophilus* DusA (PDB: 3B0V), human Dus2 without the dsRBD (PDB: 4XP7), and the dsRBD of hDus2 (PDB: 4WFT), respectively. The TIM-barrel domain (TBD) appears in teal, while the helical domain (HD) is in blue, the inserted beta-sheet in red, the connecting alpha-helix (c-αH) in green, and the dsRBD in purple. The FMN coenzyme is denoted in yellow. Above each of the structures is a scheme of the modular organization of Dus2, in which the delineation of each domain is shown.

Overall, the structural analyses of all these structures allow for the definition of a canonical Dus fold, which consists of: (i) a TIM-barrel domain on the N-terminal side carrying the active site with the FMN located in the center of the barrel; (ii) a helical domain (HD) formed by 4 helices in a bundle lying on the C-terminal side; and (iii) a linker, which connects these two domains (Figure 2A,B). 

In bacterial enzymes both the TIM-barrel and HD participate in tRNA binding [22,27]. In contrast, hDus2 was shown to carry an additional domain after the HD, namely a double-strand binding domain (dsRBD) (Figure 2B) cooperating with the TIM-barrel for tRNA recognition and binding [25]. This gain in architectural modularity is accompanied by a loss of electropositivity on the HD surface of hDus2 compared to its bacterial counterparts, which no longer fully participate in substrate binding [25,32]. The dsRBD of hDus2 is unique among members of this family as it carries a new type of N-terminal extension (NTE) [25,33]. This finding raised the possibility that this new prototype of dsRBD has evolved to specifically recognize the particular 3D-L-shaped structure of tRNAs. Indeed, compelling structural evidence, namely the crystal structure of this dsRBD in complex with a dsRNA, structural characterization of the dsRBD/tRNA complex by NMR, SAXS, and extensive mutagenesis, provided evidence that this dsRBD is specialized to recognize a tRNA substrate via its NTE [29]. The latter provides specific residues that, in combination with those on the canonical dsRBD structure, expand the RNA-binding interface, allowing the newly evolved domain to bind tRNA. These observations led us to hypothesize that perhaps the modularity acquired by hDus2 may not be an isolated case within the Dus2 family and that the tRNA recognition mechanism may have undergone various evolutionary modifications.

Using phylogenetic analysis and accurate 3D protein structure prediction, we investigate here the structural evolution of eukaryotic Dus2 to identify novel modes of tRNA binding along the evolutionary tree of life. We found that Dus2 exhibits significant structural variability beyond the level of their canonical domains. Dus2 enzymes can carry structural extensions primarily on the C-terminal side that range from simple helix acquisition to the addition of a new domain. In addition to the dsRBD, we have identified five new domains that may be present in Dus2, including zinc finger modules. More importantly, analyses of protein surface electrostatics and modeling of Dus2/tRNA complexes suggest that some of these extensions are likely involved in RNA recognition. Our study illustrates how nature opportunistically refines Dus structures by decorating the canonical fold with new structural elements that function as effectors to generate new substrate recognition units.

## 2. Materials and Methods

### 2.1. Phylogenetic Distributions across the Dus Superfamily

Using representative sequences of each Dus subfamily (i.e., Q9HGN6, Dus1 of *Schizosaccharomyces pombe*; O74731, Dus2 of *S. pombe*; Q9UTH9, Dus3 of *S. pombe*; O74553, Dus4 of *S. pombe*; P32695, DusA of *E. coli*; P0ABT5, DusB of *E. coli*; P33371, DusC of *E. coli*; Q57608, archaeal Dus of *Methanocaldococcus jannaschii*), OrthoInspector [34] were used to extract sequences of Dus homologs. OrthoInspector maintains a benchmark set of genomes, which it uses to consistently determine the absence or presence of orthologs, including both model and non-model organisms across archaea, bacteria, and eukaryota. This allows, with each query, additional lists of organisms in which homologs of the query sequence are “not present in”, as well. All lists resulting from these queries were concatenated, and redundancies were removed. The sum of organisms from both list types, “present” and “not present in” was used to derive the final list of organisms to use in these analyses (Supplemental Table S1). To confirm subfamily membership of each sequence, an arbitrary number of sequences per batch (50–100 sequences) were checked by performing sequence alignments and generating sequence trees containing positive controls for each subfamily (i.e., the controls used were equivalent to the Dus sequences used in retrieval queries) (for an example of this approach, see the Supplemental example in Figure S1). The latter was completed using ClustalO (EMBL-EBI; https://www.ebi.ac.uk/Tools/msa/clustalo/ accessed on 21 April 2022) for each checked subset [35]. The assignment of subfamily membership for each sequence was determined according to their phylogenetic proximity to the aforementioned control (i.e., query) sequences. After subgroup membership was assigned, these were then used to determine the absence or presence (counts) per subgroup and per organism. Taxonomic identifiers were mapped using the UniProt sequence entries.

### 2.2. Dus2 Fusion Analysis

Using the Dus2 sequence of *S. pombe* (O74731), the BLAST tool of OrthoMCL (release 6.10, accessed on 21 April 2022) [36] was used to gather an initial batch of Dus2 family sequences of various architectures (orthologous group: OG6_102617; 527 sequences, total). Sequences were then mapped to UniProt Accession Identifiers and taxonomic IDs using the UniProt mapping tool [37], with the total number of sequences equaling 383. The CDD batch search tool [38] was used to map recognizable domains across all sequences, assigning either the highest fidelity specific hit fusion domain or CDD clan cd02801 (DUS_like_FMN). Dus2 homolog sequences of closely related organisms were used to BLAST the genomes suspected of gene losses/oversight (i.e., Dus2 homolog is missing; check via NCBI BLAST). Dus2 family members and fusions exported from InterPro [39], distinct from the sequences already curated, were then merged with the master list (total of 390 sequences) to give Supplemental Table S2 after being verified for Dus2 subfamily membership, again using the Dus homologs 1–4 of *S. pombe* as positive controls in an alignment and then a sequence tree (example, Supplemental Figure S2). Lengths of Dus family domains were determined from UniProt domain annotation and, if UniProt annotations were lacking, CDD Search. The phylogenetic tree was generated using PhyloT (database 2022.1; https://phylot.biobyte.de, accessed on 26 August 2022) and iToL [40]. Data (i.e., sequence lengths, domain fusions) were mapped using the iToL tree editor (accessed 26 August 2022).

### 2.3. Dus2 Sequence Logos

JalView was used to perform the multiple sequence alignments (ClustalO program within JalView) for the Dus2-specific set of homologs (see Dus2 Fusion Analysis methods subsection) [41]. Sequence logos were made using WebLogo (https://weblogo.threeplusone.com, accessed on 24 August 2022) [42].

### 2.4. AlphaFold Models

All AlphaFold models were generated using AlphaFold2, which is hosted through ColabFold (https://colab.research.google.com/github/sokrypton/ColabFold/blob/main/AlphaFold2.ipynb accessed on 1 June 2022) [43]. Atomic coordinates for these files with a pLDDT score less than 70 were not analyzed. 

### 2.5. Cloning of dsRBD of Dus2 from Amphimedon Queenslandica

We obtained a commercially supplied synthetic plasmid of the dsRBD of Dus2 from *Amphimedon queenslandica* (residues 266–371) (pEX- dsRBD_Aq ampicillin-resistant) from Eurofins. We used this plasmid to amplify by PCR and clone into pET22b the gene of *A. queenslandica* Dus2 dsRBD (dsRBDAq), which contains a sequence encoding for a 6-histidine tag placed at the C-terminal region of the protein. The PCR fragment was amplified with these primers (Forward aagaaggagatatacatatgAAATCGAAAATGGATCCAGAAG; Rerverse gcggtcggcagcaggtattttcagtggtggtggtggtggtgTGAATTGCTGGCAGTTGAC) and purified with QIAquick PCR purification kit (Qiagen, Germantown, MD USA), before cloning with SLIC cloning method into pET22b previously linearized with PCR using these primers (Forward AAATACCTGCTGCCGACC; Reverse CATATGTATATCTCCTTCTTAAAGTTAAAC) and gel purified with QIAquick gel purification kit (Qiagen). Chemically competent DH5α cells were transformed with the plasmid, and gene integrity was verified by DNA sequencing (Eurofins).

### 2.6. Overexpression and Purification of dsRBDAq 

Overexpression of dsRBDAq was achieved using a chemically competent BL21 (DE3) *E. coli* strain (Novagen, Madison, IA USA) transformed with pET22b-dsRBDAq. 100 mL (LB medium) of the overnight cultures were used to inoculate a larger scale cell culture (6 L) at 37 °C until the optical density at 600 nm reached 0.5. Protein synthesis was induced by the addition of Isopropyl 1-thio-β-Dgalactopyranoside to a final concentration of 1 mM. Cells were grown for an additional 16 h at 16 °C, collected by centrifugation (9000× *g* at 4 °C for 10 min), and stored at −80 °C until use. Cells were resuspended in 50 mM Tris-HCl pH 8, 500 mM NaCl, 10% glycerol, 5 mM imidazole, and 1% triton X-100 and discontinuously sonicated for 15 min in a water-ice batch. Cellular extracts were centrifuged for 45 min at 193,000× *g*, which yielded a soluble fraction of dsRBDAq.

The resulting supernatant was loaded onto a Hitrap Excel column (5 mL, GE Healthcare) equilibrated with buffer, 50 mM Tris-HCl pH 8, 300 mM NaCl, 10% glycerol, and 5 mM imidazole. Bound proteins were eluted with a gradient of imidazole (0–500 mM) in buffer: 50 mM Tris-HCl pH 8, 300 mM NaCl, 10% glycerol, and 500 mM imidazole; dsRBDAq was eluted at 150 mM imidazole. SDS- PAGE allowed the identification of dsRBDAq, and the purest protein was pooled and concentrated with Amicon Ultra 10K cut-off concentrators (Millipore) until a volume of 5 mL was reached. 45 mg of protein was loaded onto a HiLoad 16/600 Superdex 75 pg equilibrated with 50 mM Tris-HCl, pH 8, 300 mM NaCl, and 5 mM 2-Mercaptoethanol. Protein concentrations were determined by a Bradford assay (Bio-Rad), with BSA used as a standard. The elution volume of dsRBDAq was 78 mL. The pure and monomeric protein fractions were pooled, concentrated to 11 mg/mL, frozen in liquid N2, and then stored at −80 °C.

### 2.7. Crystallization, Data Collection, and Structure Determination 

Crystals of dsRBDAq were grown by the hanging drop vapor diffusion method at 292 K by mixing the purified dsRBDAq (at 15 mg/mL in 50 mM Tris, pH 8, 300 mM NaCl, and 5 mM β mercaptoethanol) with an equal amount (1 μL) of reservoir solution (0.1 M Hepes sodium salt, pH 7.5, and 1.4 M tri-sodium citrate as precipitant) and seeds previously prepared. After a few days, crystals appeared and were swept through a reservoir solution complemented with 30% glycerol and immediately flash-frozen in liquid nitrogen. X-ray diffraction data were collected at the synchrotron SOLEIL on the beamline Proxima 2 and were indexed, integrated, and scaled using the autoPROC pipeline [44] (ref). The structure was solved by molecular replacement with phenix.phaser [45] using a search model processed with sculptor [46] and based on 4wft. Phases and models were improved with phenix.autobuild [47]. The final model and phases were obtained by alternating manual building in Coot [48] and refinement in BUSTER (www.globalphasing.com/buster/ version v2.10.4 (8-JUN-2022)). Data collection and refinement statistics are shown in Table 2.

## 3. Results and Discussion

### 3.1. Phylogenetic Distribution of Dus2 and Domain Analyses

To understand the distribution and more readily interpret the plausible evolutionary history of Dus2, a benchmark subset of 324 genomes were investigated for the absence or presence of all Dus family subgroups, both derived from OrthoInspector (see methods and Supplemental Data Table S1). In batches of 50–100 sequences, homologs were checked for subgroup membership through multiple sequence alignments and sequence trees, including described control sequences (see methods for sequences; example of approach, Figure S1). Approximately 41% of organisms considered in this study were found to possess at least one Dus2 family member, making it the third most common of all Dus subfamilies. Similarly, 40% of taxonomic groups (see partitions and groups in Supplemental Data Table S1) were observed to have at least one organism possessing Dus2. As anticipated, organisms found to be without a single Dus2 homolog included all archaeal taxonomic groups (i.e., Asgard Group, DPANN, Euryarchaeota, TACK Group) and all bacteria (i.e., FCB Group, Proteobacteria, PVC Group, Terrabacteria Group, Other Bacteria) (Supplemental Data Table S1). Of organisms with Dus2 and another of a different Dus family subgroup, they were more likely to also have a Dus3 homolog than any other Dus subfamily member (Supplemental Data Table S1). 

**Figure 3:**
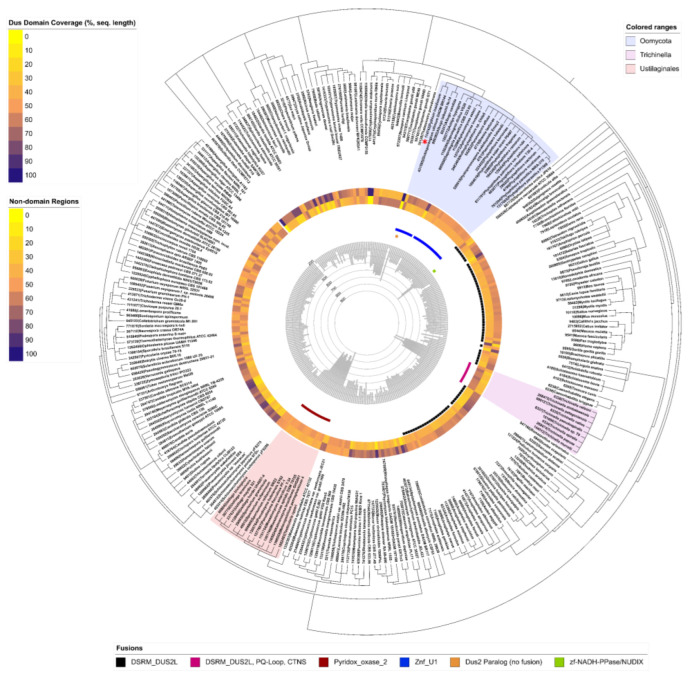
Phylogenetic tree of 280 Dus2 family members illustrating domain architecture diversity. Moving from the inside of the figure to the outside: (1) Sequence lengths are denoted by the central histogram; (2) Colored bars (one square per organism) indicate the presence of distinct fusion proteins (note: given multiple squares for one organism, they indicate the instance of separately encoded homologs); (3) Percent coverage of sequences for both the remaining lengths not identified as belonging to any recognizable domain (inside) and the identified Dus domain (outside) are shown. A red asterisk among the labels of the tree’s leaves denotes the zinc finger domain-containing homolog of *Pythium insidiosum* that, in addition to ZnD_U1, also contains a separate ICL_KPHMT fusion domain.

To better understand the within-family diversity of Dus2, domain architectures and fusions were collected from various functional annotation databases. Using a Dus2 control sequence of *S. pombe*, a BLAST query of OrthoMCL was used to determine a precise Dus2 orthologous group (OG6_102617; 527 sequences). Subsequently, the InterPro and Pfam databases were used to acquire any additional unique architectural variants of Dus2, all of which were confirmed, individually, as proper subfamily members by using the four *S. pombe* control sequences (Dus1–4) in alignments and sequence trees (Supplemental Figure S2). Ultimately, these sequences were concatenated into a Dus2 master list of 390 sequences (Supplemental Data Table S2). Using CDD batch search, all sequences were assigned a name referring to the specific hit domains present. These fusion “names” were then used in the binary determination of absence or presence for unique fusions per organism (Figure 3). 

The sequence lengths, domain lengths, and occurrence of N- and C-termini (binary) were acquired (Supplemental Data Table S2). Across all Dus2 sequences, the average sequence size was found to be 379 amino acids in length, with a maximum length of 793 aa and a minimum of 154 aa (Supplemental Data Table S2; Figure 3). In examining the diversity within the Dus2 subfamily, six unique domains were found to be fused with Dus2 domains (Figure 3): ZnF_U1 (smart00451, PSSMID 197732), ZnF_U1 + ICL_KPHMT (cd00377, PSSMID 119340), DSRM_DUS2L (cd19871, PSSMID 380700), zf-NADH/PPase/NUDIX (PF09297, PSSMID 401294), DSRM_DUS2L + [PQ-loop(x2)] + CTNS (PF04193, PSSMID 398045; smart00679, PSSMID 128923), and Pyridox_oxase_2 (PF12766; PSSMID 403846). The first two are combined in labeling within Figure 3, as the second fusion of the two was only found in a single sequence. Fusion domains, if present, were always found to occur within the C-terminal region of each sequence. With such notable levels of diversity, it was necessary to perform further structural analyses of Dus2 hybrid proteins.

### 3.2. Structural Analysis of the Dus2 Canonical Domains

**Figure 4:**
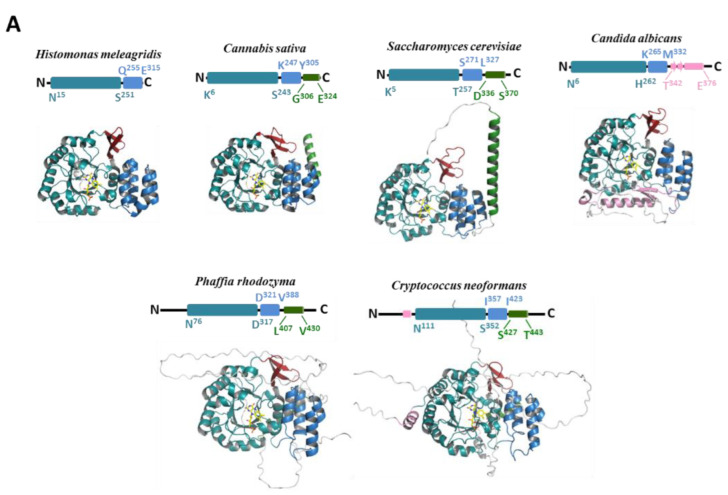

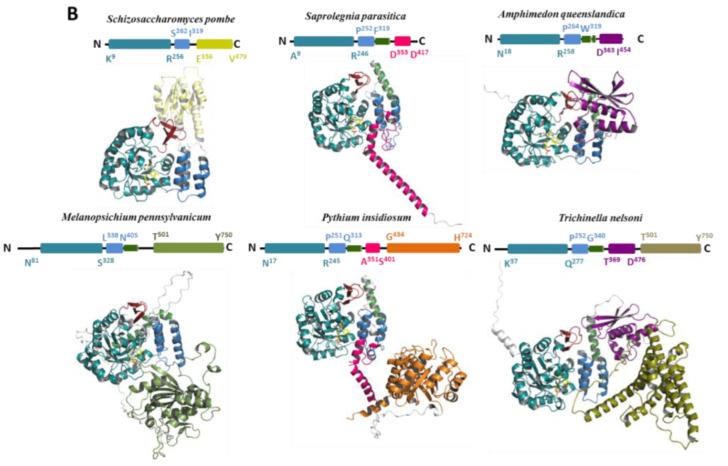
3D structural models of various Dus2. (**A**) Models of Dus2 showing minimal modularity. The TIM-barrel domain (TBD) appears in teal, while the inserted beta-sheet is in red, the helical domain (HD) is in blue, the connective alpha-helix (c-αH) is in green, and the other structural extensions are in pink. The FMN coenzyme is denoted in yellow. (**B**) Models of Dus2 showing complex modularity with the addition of an extra domain. The same color codes for the canonical domains are followed (TBD + inserted beta sheet + HD). Rossman, zinc finger, dsRBD, PyrOX_2, ICL_KPHMT, and CTNS domains are in yellow, pink, purple, light green, orange, and olive, respectively. Above each of model is represented the schematic modular organization of Dus2 and is indicated the boundary of each domain. We have chosen not to show the delineation of the inserted beta-sheet in order to avoid figure overload. However, this structural element is colored in red in each of the 3D models presented.

Of the 30 structural models of Dus2 calculated along the evolutionary tree from bikonts to animals, all proteins analyzed retained the canonical folding, i.e., the TIM-barrel on the N-terminal side followed by the HD domain (Figure 4 and Figure S3). The per-residue confidence score (pLDDT) values produced by AlphaFold2 for these two domains are very high, generally above 90, indicating strong confidence in the structural patterns of these regions. In addition to these domains, there are N-terminal and C-terminal extensions of proteins that we will discuss in the following section (see below).

#### 3.2.1. Catalytic Domain (TIM-Barrel)

When the AlphaFold models are structurally aligned with the 4XP7 or 4WFS crystallographic structures, the RMSD values are all less than one. For example, the RMSD values obtained for *Candida albicans* Dus2 versus 4XP7, *Cryptococcus neoformans*, *S. pombe*, *Aspergillus awamori*, *Fusarium oxysporum,* and *S. cerevisiae* are 0.886 (over 202 atoms), 0.855 (over 223 atoms), 0.801 (240 atoms), 0.783 (231 atoms), 0.713 (227 atoms), and 0.702 (202 atoms), respectively (Figure S4). The N-terminal region of Dus2 consists of a conserved α11/β11 TIM-barrel fold, in which a central barrel composed of eight parallel beta strands is surrounded by 11 alpha helices (Figure 2B and Figure 4). This structural arrangement is partly reminiscent of that found in flavoproteins such as dihydroorotate dehydrogenase and domain IV of dihydropyrimidine dehydrogenase, enzymes catalyzing comparable reactions [49]. As expected for α/β-barrel flavoenzymes, the central barrel offers a cavity for the binding of the redox coenzyme, FMN. The TIM-barrel core diverges from the classical α8/β8 fold because three additional β-strands insert at the N-terminal region of the TIM-barrel forming a new antiparallel β-sheet (β3-β5) (Figure 2B and Figure 4), which seems strictly conserved among the Dus2 family. For example, this β-sheet inserts between C46 and Y76 in *S. cerevisiae*, between S46 and H77 in *C. albicans,* and between T49 and H76 in *S. pombe*. This structural element was first reported in hDus2 (Figure 2B) [25]. However, it was thought to be a peculiarity of hDus2 because it was not present in the crystallographic structures of DusA, DusB, and DusC (Figure 2A). Thus, we can clearly identify this β-sheet as a distinguishing feature of the canonical Dus2 fold. Note that this distinctive additional β sheet has not been predicted by the previous homology model of Dus2 [20].

#### 3.2.2. Active Site

Since AlphaFold was recognized to predict all-atom accuracy of 1.5 Å rmsd95 compared to experimentally determined structures meaning that it can also produce highly accurate side chains [50], we decided to examine Dus2 active sites. The models produced were carried out on the apoprotein, although Dus2 is a flavoenzyme that uses FMN as a prosthetic group. In order to examine the active site in more detail, we superimposed the calculated models on the 4XP7 or 4WFS crystallographic structure. It is interesting to note that, first of all, none of the side chains of the models clash with the FMN present in the crystallographic structure of hDus2. Moreover, most of the conserved residues adopt a conformation similar to those observed in 4XP7. Thus, the analysis of the active sites does not seem to be biased by the fact that one is examining an apoprotein model. The resulting structural superpositions for three Dus2 are shown in Figure S5. As expected, the FMN cofactor lies inside a deep, positively charged crevice, stabilizing the negative charge of the phosphate moiety of the cofactor. All the constituting parts of the FMN, i.e., the isoalloxazine ring and the ribityl phosphate chain, make extensive interactions with surrounding amino acid residues, ensuring a tight binding and a proper orientation of the entire coenzyme. The strictly conserved Met (M19 in hDus2, M13 in *S. cerevisiae* and *C. albicans*, and M15 in *S. pombe*) stacks against the re-face of the isoalloxazine ring and is placed at ∼4 Ǻ from the FMN C8 carbon. The side chains of three extremely conserved residues, a Q, N, and K (Q87/N113/K155 in hDus2, Q88/N114/K160 in *S. cerevisiae*, and Q87/N113/K159 in *C. albicans* and *S. pombe*, Supplemental Figure S6), interact with the pyrimidine moiety of FMN. The specifically positively charged lysine residue in this triad is likely to stabilize the negative density of FMNH^-^ (Figure S7) obtained upon flavin reduction by NADPH. A 13-amino-acid loop spanning residues 116 to 128 and acting as a lid on the active site and inserted in the TIM-barrel between β6 and the small α5 helix is disordered in the crystal structures of hDus2. This loop contains the highly conserved C116 for hDus2, which has been proposed to function as a proton exchange site [2,18,22]. The corresponding loop gets ordered in the presence of tRNA [22]. Interestingly, in all the models, this active site loop appeared structured, forming a short eight-residue alpha-helix. While this cysteine is oriented away from the active site in the crystallographic structures of hDus2, in the models, this key residue (C116 in *S. cerevisiae*, *S. pombe,* and C117 in *C. albicans*) faces the flavin by being positioned above the isoalloxazine.

#### 3.2.3. Helical Domain

The TIM-barrel is connected to the HD by a short linker whose length varies within the Dus2 family. For example, this linker is composed of thirteen residues in *S. cerevisiae* (T257 to D268), *H. meleagridis* (S251 to K256), *S. pombe* (R256 to S262), six in *H. sapiens* (L253 to R258) based on X-ray crystallography, and in *S. parasitica* (R246 to L251), whereas in *C. sativa*, it is composed of five amino acids (S243-K247). The helical subdomain is formed by a bundle of four helices, a feature that also appears to be conserved in bacterial Dus [19,22,26,27]. The TIM-barrel and HD domains of all Dus2 share a predominantly hydrophobic interaction surface but stabilized by additional electrostatic contacts, including hydrogen bonds and π-cationic interactions as observed in the crystallographic structure of the canonical domains of Dus2. The aromatic residues present in the interface may participate in the orientation of the HD relative to the catalytic domain. The relative orientation of these two domains is globally conserved among Dus2, as shown by the structural alignments between the 4XP7 and Dus2 models (Figure S4). However, this orientation differs from that of DusA, which is the bacterial homolog that, like Dus2, catalyzes D20 formation (Figure 2A). The presence of the additional β-sheet (β3-β5) that is inserted into the TIM-Barrel of Dus2, increases the surface area of interaction between the TIM-Barrel and the HD compared to that of bacterial Dus. On the basis of the crystallographic structures of hDus2, the increase in this interface was estimated to be 15% of the surface area [25]. Although globally the HD is well conserved in Dus2, we noted that the loop connecting helix 2 to helix 3 may exhibit size variability. While this loop consists of 5 residues in humans (R290-E294), it extends to 11 residues in *S. parastica,* for example, making it 16 residues long (S284-D299).

### 3.3. Structural Extensions of Dus2

Beyond the canonical Dus architecture, many Dus2s carry N- and C-terminal extensions or only an N-terminal or C-terminal extension (Figure 4). Some Dus2s such as *H. meleagridis,* have no extensions, suggesting that there are Dus2s that can obviously function only with the TIM-barrel and HD domains like in the bacterial Dus. In Dus2, extensions can be classified into two types: (i) unstructured sequence additions; (ii) minimal order structural extensions such as the addition of a helix, such as the connecting α-helix (c-αH) placed just after the HD (see below) or other structural elements that do not constitute a protein domain. 

#### 3.3.1. Unstructured Extensions 

Structureless extensions can be observed at both ends of the protein (Figure 4A,B). The size of these extensions can vary from a few amino acids to much longer lengths, as is the case, for example, with Dus2 from *C. neoformans,* which has an unstructured extension of 80 amino acids at the N-terminus (M1 to S81) but also another at the C-terminus of about 50 amino acids (R445 to S494) (Figure 4A). All the analyzed unstructured extensions showed pLDDT values <50. The five models proposed by AlphaFold (ranked by score) for each of the Dus2, present extensions that adopt several positions in space. The lack of structure could be in agreement with a sampling of the conformations, as seen with the intrinsically disordered proteins (IDP), or it could be that AlphaFold is not able to predict a structure for these regions because it did not find interchain contacts due to its intrinsic limitation. However, the first explanation appears to be the most convincing. This is supported by structural evidence obtained from the C-terminal region of mouse Dus2. Indeed, a PDB of the solution structure of the isolated dsRBD domain of mouse Dus2 under the code 1WHN and annotated “Solution structure of the dsRBD from hypothetical protein BAB26260” is available [2]. In this NMR models, the long C-terminal extension is disordered likely due to the lack of restraints, a consequence of the intrinsic flexibility of this region. Hence, low-confidence residues may be explained by some form of disorder, although one should be cautious about it. Indeed, IDPs are common in the proteomes of eukaryotes, and a study estimated that the percentage of disordered residues in the human proteome is between 37% and 50%. These disorder predictions could also encompass both regions that are intrinsically disordered and regions that are structured only in complexes with cellular partners [51].

#### 3.3.2. Structured Extensions and the Connecting α-Helix

Some structured extensions show good pLDDT scores. The HD is very often followed by an additional long alpha helix, as in the case of 27 out of 30 generated models, including S. *cerevisiae* for example, suggesting the important conservation of this specific structural entity of Dus2 (Figure 4). Among the thirty Dus2 models generated, only *H. meleagridis*, *C. albicans,* and *S. pombe* Dus2 lack this helix. This helix can be fragmented into two helices as in Dus2 of *F. oxysporum* and *A. awamori,* for example. We propose here to name this helix the connecting helix (c-αH), as it connects the additional domains (see below) to the canonical domains. We and Antson’s group had observed the presence of this helix in hDus2, which is absent in bacterial Dus, but it was not clear whether this was specific to the human enzyme [25,28]. It can now be stated that c-αH is a characteristic feature of Dus2 proteins. The overall good results of the *S. cerevisiae* model, including the c-αH, allow us to analyze this extension in more detail. This helix begins with residue D336 and ends with S370 (Figure 4A). Indeed, the prediction of the c-αH of about 30 residues is robust, as shown by the scores (>90 between P337-N353, between 90 and 70 for residues from A354 to Q364) (Figure S8). C-αH is attached to the HD by a linker of ten amino acids. In a sense, this alpha helix extends the HD on the opposite side of the TIM-barrel. The orientation of c-αH is maintained by ionic and hydrophobic interactions engaging its N-terminal region with certain residues of helices-3 and -4 of the HD. C-αH is followed by fourteen unstructured amino acids (S370-I384), with I384 being the last residue of the protein. 

Beyond the c-αH, other structural extensions may exist. For instance, the extension observed in *C. albicans* Dus2 is very unique because it includes several novel structural elements and does not carry the c-αH (Figure 4A). First, the HD helix-4 is elongated by thirteen residues (K320-Q331) projecting down the domain, in the opposite direction to the c-αH observed in *S. cerevisiae* Dus2. This helix is attached to a beta-sheet formed by 2 strands by a linker. This beta-sheet is itself linked to a long alpha helix of 23 amino acids. These novel structural features extend the TIM-barrel on the proximal side and are stabilized by numerous interactions between the TIM-barrel and the HD domains. As with *S. cerevisiae* Dus2, the structural extension ends with an unstructured region of approximately fifty residues. Similarly, hDus2 ends with an unstructured region, which appears to be often the case in Dus2 enzymes.

### 3.4. Modularity of Dus2

**Figure 5:**
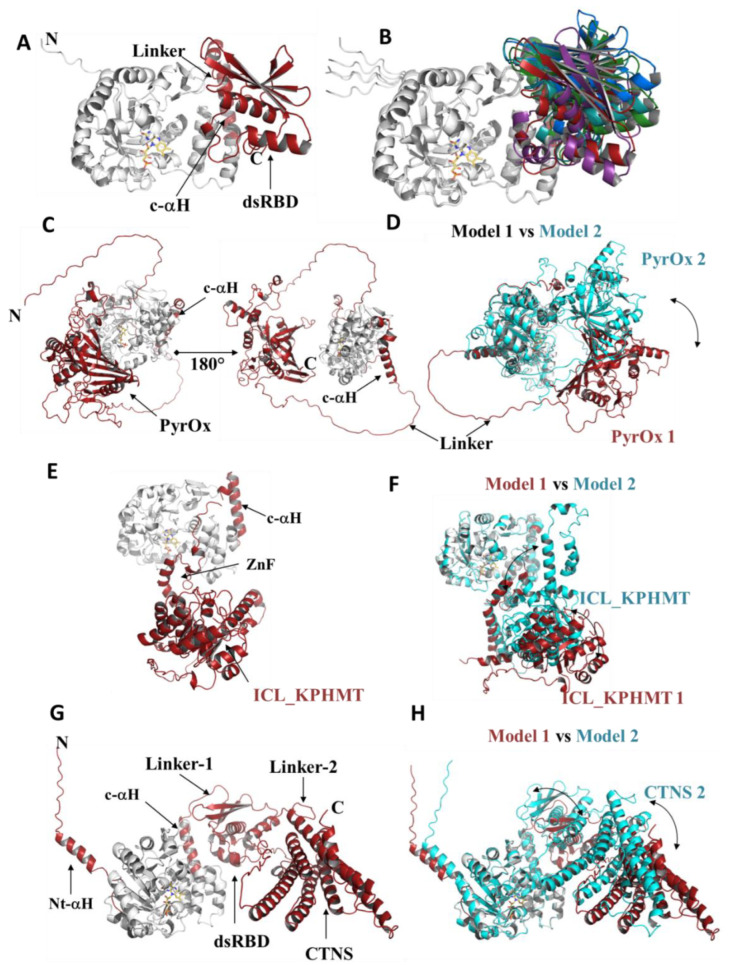
Modularity and domain orientation in Dus2. (**A**) Structural 3D model of *A. queenslandica* Dus2. The canonical domains are in gray while the c-αH and the dsRBD are in red. The linker attaching the c-αH to the dsRBD is indicated. (**B**) Structural superposition of the 5 *A. queenslandica* Dus2 model generated by AlphaFold2. The TIM-barrel and the HD of each model are in gray while the dsRBD are colored in a different color for each model. (**C**) Structural 3D model of *M. pennsylcanicum* Dus2 showing to different view that differs by a rotation of 180° around the z-axis. The canonical domains are in gray while the c-αH and PyrOx domains are in red. (**D**) Structural superposition of models 1 and 2 of *M. pennsylcanicum* Dus2 generated by AlphaFold2. Model 1 is colored as indicated in (**C**) while model 2 is in cyan. The double-headed arrow indicates the different orientation of the PyrOx domain in the two models. (**E**) Structural 3D model of *P. insidiosum* Dus2. The canonical domains are in gray while the c-αH, the ZnFD, and the ICL-KPHMT domains are in red. (**F**) Structural superposition of models 1 and 2 of *P. insidiosum* Dus2 generated by AlphaFold2. Model 1 is colored as indicated in (**E**) while model 2 is in cyan. The double-headed arrows indicate the difference in orientation of the ZnFD and ICL-KPHMT domains in the two models. (**G**) Structural 3D model of *T. nelsoni* Dus2. The canonical domains are in gray while the c-αH, the dsRBD, and the CTNS domains are in red. (**H**) Structural superposition of models 1 and 2 of *T. nelsoni* Dus2 generated by AlphaFold2. Model 1 is colored as indicated in (**G**) while model 2 is in cyan. The double-headed arrows indicate the respective difference in orientation of the dsRBD and CTNS domains in the two models.

Beyond simple structural extensions, some Dus2 have an entire domain appended to the canonical domain. Our phylogenetic analysis identified five domains present in Dus2 (Figure 3). However, we were able to identify an additional domain by modeling Dus2 with sizes larger than those of a protein containing only the canonical domains (between 300 and 320 amino acids), a domain that had not been picked up by phylogenetic analysis alone (Figure 4B, case of *S. pombe*). All these domains are always added after the HD, and more particularly after the c-αH when this helix is present. Although for the majority of Dus only the addition of a single domain is observed, there are very few cases where we find the grafting of two additional domains to the two existing canonical domains, as is the case, for example, for *P. insidiosum* or *Trichinella nelsoni* (Figure 4B). These added domains are generally attached to the rest of the protein by long, flexible linkers that provide these modules with a large degree of freedom (Figure 5). This is perfectly illustrated on the different models generated because, for a given Dus2, the positioning of these domains is generally not conserved and thus orients differently from one model to the next due to the absence of inter-domain contacts found by AlphaFold2 (Figure 5). Aside from the zinc finger domain (ZnFD) and the double stranded RNA binding domain (dsRBD), which are well known for their ability to bind dsRNAs, the involvement of the other domains in RNA and nucleic acid metabolism in general has not been documented. It should be noted that we were not able to obtain a reliable model of the nudix domain due to the very low scores.

#### 3.4.1. Zinc-Finger Domain

**Figure 6:**
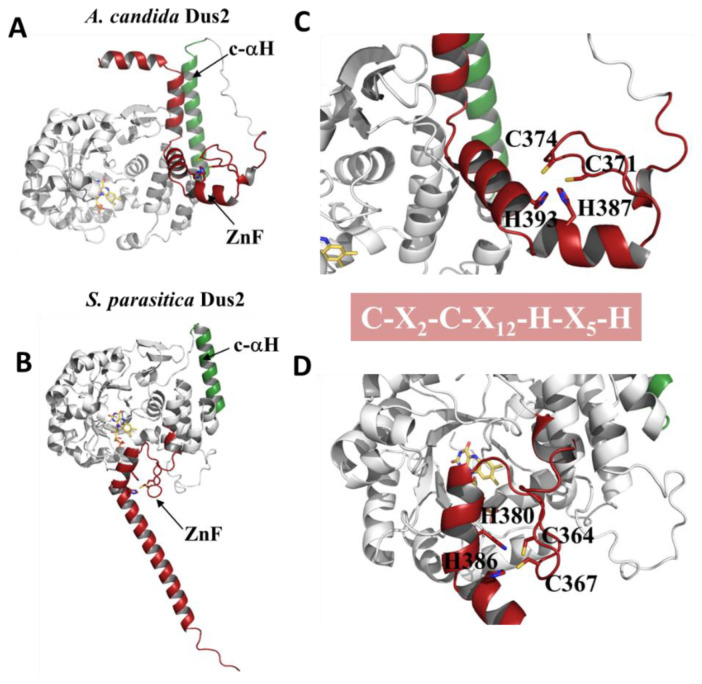

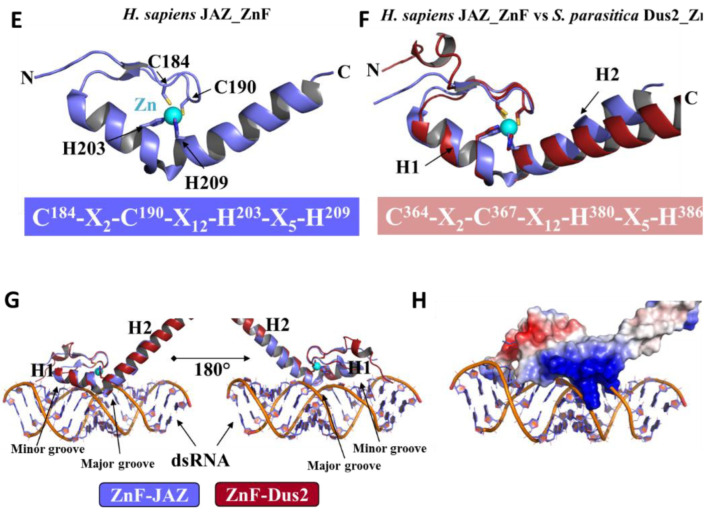
The Zinc finger domain in Dus2. (**A**) Structural 3D model of *A. candida* Dus2. The canonical domains are in gray while the c-αH and the ZnFD are in green and red, respectively. (**B**) Structural 3D model of *S. parasitica* Dus2. (**C**,**D**) Zoom on the zinc finger motifs of *A. candida* and *S. parasitica* Dus2, respectively. The signature of the motif is indicated in the red box. (**E**) Solution NMR structure of a ZnFD of human JAZ protein (PDB: 2MKN). The zinc atom is represented as a ball colored in cyan. The ZnF motif for this domain is indicated below the structure. (**F**) Structural superposition of 2MKD (colored in purple) with the ZnFD of *S. parasitica* Dus2 (in red). The ZnF motif of *S. parasitica* Dus2 is shown below the figure of the structural alignment. (**G**) Structural superposition of 2MKN (PDB code for the structure of JAZ ZnFD in complex with a dsRNA with the ZnFD of *S. parasitica* Dus2 (in red). Two different views that differ by a rotation of 180° around the z-axis are shown. (**H**) Model of the *S. parastica* Dus2/dsRNA complex. The ZnFD is represented in the electrostatic surface mode.

The ZnFD follows the c-αH, the latter acting as a connector between the HD and the ZnFD. The size of this domain is globally conserved; e.g., *S. parasitica* has a ZnFD of about 64 amino acids in length. In all cases, this domain serves as the C-terminal domain, except in the case of *P. insidiosum,* where the ZnFD is followed by an additional ICL-KHMPT-like domain (Figure 4B). The ZnFD of Dus2 consists of a 3–10 helix followed by a 2-stranded beta-sheet that takes the form of a finger, and then comes a first helix (H1) with two turns, a kink, and a second larger helix (H2) (for example, nine turns for the case of *S. parasitica*). The positioning of this ZnFD relative to the rest of the protein can vary between Dus2 models. To illustrate this, we present the Dus2 of *A. candida* and *S. parasitica*. As shown in Figure 6, the *A. candida* ZnFD is facing the c-αH extension (Figure 6A), whereas in the case of *S. parasitica*, the ZnFD is positioned in front of the TIM barrel and the HD (Figure 6B). It seems therefore difficult to determine the exact location of the domain, however it is quite possible to imagine that this ZnFD may have a degree of freedom allowing it to move and adapt its position in particular in the presence of the RNA substrate thanks to a long linker of about 15 amino acids that attaches this domain to the HD. The ability to move is a known property of ZnFDs. The ZnF motif of Dus2 is of the CX2CX12HX5H type, part of the large family of C2H2 class zinc fingers, the most commonly used in transcription factors with the ability to bind preferentially to double-stranded RNA (dsRNA) [52,53]. 

The two cysteines of the motif are present in the central beta-sheet while the two histidines are found in each of the two helices, H1 and H2 (Figure 6C,D). Interestingly, the ZnFD of Dus2 shares a similar structure to the zinc fingers of the human Just Another Zinc finger (JAZ) protein [54] (Figure 6E). This four zinc finger protein is known to bind endogenous and exogenous dsRNAs such as adenoviral VAI RNA [54]. Indeed, superimposing the ZnFD of Dus2 *S. parasitica* on the NMR structure of JAZ ZnFD in the 2MKD PDB gives a very low RMSD (~0.696 Å over 25 Cα) consistent with the structural conservation of this domain in the 2 proteins (Figure 6F). Even more, the side chains of the ligands of the zinc atom, namely C2H2, adopt perfectly identical orientations to those of JAZ, suggesting that these residues are oriented in their functional form in the models generated by AlphaFold (Figure 6F). Structural alignment of the ZnFD of Dus2 *S. parasitica* with the NMR structure of the JAZ:dsRNA complex (PDB: 2MKN [54]) shows that the two proteins share the same dsRNA recognition mode (Figure 6G). Indeed, dsRNA binding occurs mainly via H1, kink, and H2. H1 and H2 recognize the minor grooves, while the kink and the N-terminus of H2 recognize the major grooves. Interactions between dsRNA and the ZnFD domain of Dus2 are largely driven by interactions of an electrostatic nature, as shown in Figure 5H. Specifically, H1 and H2 have charged residues that will generate a highly positively charged surface to accommodate phosphates on the RNA backbone. In JAZ, the dipole moment of H2 also contributes to this interaction [54].

**Figure 7:**
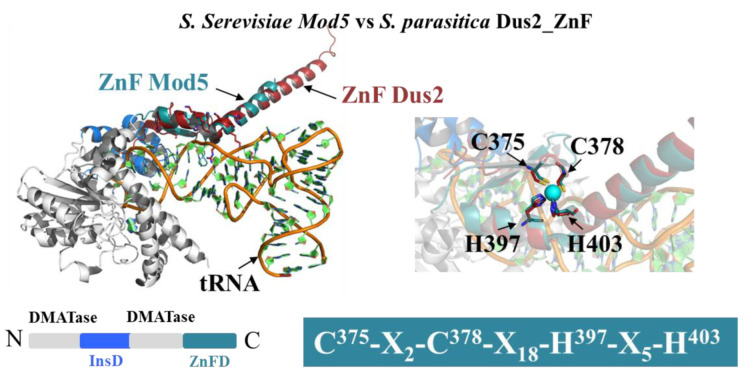
Structural comparison of the ZnFD of Dus2 and that of *S. cerevisiae* Mod5. ZnFD of *S. parasitica* Dus2 (in red) is superimposed on the ZnFD of Mod5 (in deep teal color) in the crystal structure of yeast Mod5/tRNACys (PDB: 3EPH). The below the figure is the schematic representation of the domain modularity of Mod5. The catalytic, inserted, and ZnFD of Mod5 are in gray, blue, and deep teal, respectively. A zoom of the superposition on the ZnF motif region is shown on the right. The ZnF motif of Mod5 is indicated below the zoom in the deep teal colored box.

The portion of the tRNA that will be recognized by the Dus2 ZnFD remains undetermined at this stage. However, it should be noted that the tRNA contains many double-stranded, structured regions. In addition, there are RNA modification enzymes that also use ZnFDs as additional tRNA recognition modules. One example is Mod5, an isopentenyl transferase that catalyzes the formation of i6A37 in *S. cerevisae* tRNAs, and that in addition to its catalytic domain and insertion domain has a C2H2-type C-terminal ZnFD that recognizes the tRNA anticodon stem as shown in the crystal structure of Mod5:tRNACys [55] (Figure 7). Similar to JAZ, the ZnFD of Dus2 from *S. parasitica* overlaps with the ZnFD of Mod5 except for the central beta-sheet. However, despite this discrepancy, the C2H2 residues of Dus2 and Mod5 overlap perfectly and adopt an identical orientation with respect to the zinc atom. Furthermore, no clash was detected between the ZnFD of Dus2 and tRNA.

#### 3.4.2. The Double-Stranded Binding Domain

The other domain acquired by more evolved Dus2 is the dsRBD (Figure 4B and Figure 5A). Initially observed in human Dus2, a phylogenetic analysis showed that the dsRBD is indeed more widely distributed in nature than expected since it is present in Dus2 of animals [20]. However, where exactly this additional domain appeared in the phylogenetic tree of Dus2 remains enigmatic. By reanalyzing the sequences of eukaryotic Dus2, we found out that the dsRBD does not seem to be restricted to animals but is also present in Choanoflagellates and in Filasterea, all of which are part of the Filozoa clade. A more rigorous search of the sequences allowed us to trace the phylogenetic tree and show that an Ichthyosporea, *S. arctica*, and a Cristidiscoidea, *F. alba* also possess a Dus2 with a dsRBD. We produced seven models of Dus2 carrying dsRBDs, two in animals (hDus2 and Dus2 from *A. queenslandica*), two Choanoflagellates, one Filastera, one Ichthyosporea and one Fonticuli. Interestingly, the genome of *C. owczarzaki* was found to have two paralog fusions: one containing the dsRBD domain and the other containing the aforementioned zf-NADH/PPase/NUDIX (PF09297, PSSMID 401294) domain. The former, typically around 68 amino acids, is well-known for its functional versatility by means of a particular α1-β1β2β3-α2 canonical structure that allows the recognition of a variety of simple RNA structures ranging from A-form RNA helices to hairpins or tetraloops in shape-dependent manners [56,57,58], even though a sequence-specific mode of recognition has been invoked for a few of them [59,60]. We showed that the dsRBD of hDus2 has an additional extension at the N-terminal, named NTE, which is also involved in the binding of dsRNA and specifically the acceptor-TΨC stem region [29], which is the longest dsRNA region of the tRNA. 

The models obtained for the various full lengths of Dus2 show that the dsRBD is attached to the HD indirectly via a c-αH like that identified in *S. cerevisiae* Dus2. However, a notable difference lies in the fact that in dsRBDs-containing Dus2, c-αH protrudes outside the plane formed by the HD domain so that it points towards the back of the protein. This difference in orientation could be concomitant with the acquisition of the dRBD in order to properly position the dsRBD. The orientation of the dsRBD relative to the rest of the protein is not conserved within the five models proposed by AlphaFold2 (Figure 5B). We had previously shown that in hDus2, the dsRBD is connected to c-αH by a flexible linker whose role could be the adjustment of the dsRBD functional position in the presence of the tRNA substrate [25]. 

**Figure 8:**
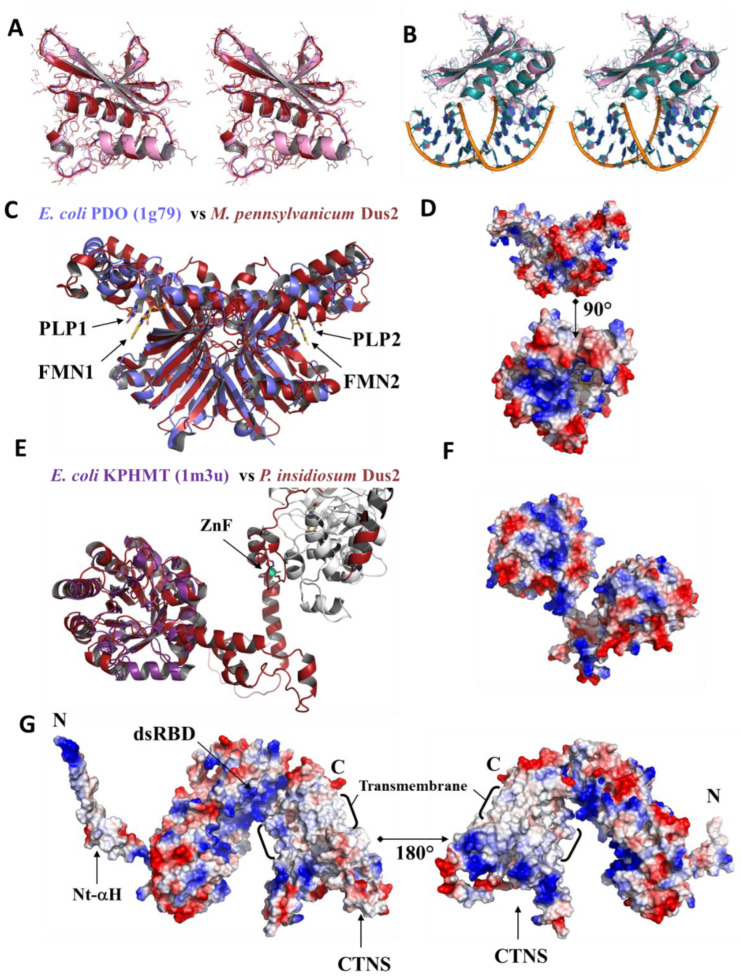
Structural analysis of various Dus2 domains. (**A**) Stereoview of the structural superimposition of the crystal structure of the dsRBD of *A. queenslandica* Dus2 (colored in pink) with the dsRBD obtained from the 3D model of *A. queenslandica* Dus2 (colored in red) generated by AlphaFold2. The side chains are shown as lines. (**B**) Stereoview of the structural superimposition of the crystal structure of the dsRBD of *A. queenslandica* Dus2 (colored in pink) with the crystal structure of the hDus2 dsRBD (colored in deep teal) in complex with a dsRNA (PDB: 5OC6). The backbone of the dsRNA is orange, while the nucleosides are deep teal. (**C**) Structural superimposition of the PyrOx domain of *M. pennsylvanicum* from the Dus2 model with the X-ray structure of the dimer of *E. coli* pyridoxine 5′-phosphate oxidase complexed with pyridoxal 5′-phosphate (PLP) and flavin mononucleotide (FMN) (PDB: 1G79). The dimer of PyrOx generates two equivalent active sites, each containing a PLP and an FMN. PyrOx of Dus2 and 1G79 are in red and purple, respectively. The FMN and PLP represented as sticks are in yellow and purple, respectively. (**D**) Electrostatic surface of PyrOx domain of *M. pennsylvanicum* Dus2. (**E**) Structural superimposition of the KPHMT domain of *P. insidiosum* from the Dus2 model (in red) with the crystal structure of ketopantoate hydroxymethyltransferase complexed the product ketopantoate (PDB: 1M3U, colored in violet). (**F**) Electrostatic surface of the KPHMT domain of *P. insidiosum* from the Dus2 model. (**G**) Electrostatic surface of Dus2 model from *T. nelsoni*.

A focus on specifically the dsRBD revealed that the overall structure is conserved among all the models. To determine if the dsRBDs of animals at the bottom of the evolutionary tree retain the same double-stranded RNA recognition mechanism compared to the dsRBD of hDus2, we crystallized the dsRBD of Dus2 from *A. queenslandica*, *Monosiga brevicolis,* and *F. alba*. Unfortunately, only *A. quenslandica* dsRBD led to diffracting crystals that allowed structural resolution. Interestingly, the validity of these models is supported by the superposition of the crystallographic structure of *A. quenslandica* dsRBD, which we solved at 1.68 angstroms in this study, with the dsRBD coming from the model (RMSD = 0.426 over 78 atoms) (Figure 8A). It is equally amazing to see that even the orientations of the side chains observed in the crystal structure are globally preserved in the model (Figure 8A). To investigate if these newly identified dsRBDs present functional similarities with those of hDus2, we structurally aligned them with the crystal structure 5OC6, which is the crystal structure of hDus2′s dsRBD (construct T339-K451) in complex with an eleven palindromic oligo-ribonucleotide that we have recently published (Figure 8B) [29]. Again, no major structural clashes can be detected between the dsRNA and the bound dsRBD. We showed that recognition of dsRNA is essentially achieved via three major canonical regions, namely helix-α1, helix-α2, and the C-terminal part of the β1–β2 loop of the dsRBD. These regions are all present in all dsRBDs analyzed here. In the case of hDus2, we showed that three residues of helix-α1 (T369, E376, and R379) interact with ribose 2′-OH groups in dsRNA’s minor groove, while K371 of helix-α1 together with K419, K420, and Q424 located in the N-terminal extremity of helix α2 recognize exclusively the phosphodiester backbone of the major groove. The C-terminal part of the β1–β2 loop binds to the minor groove via R397, which makes hydrogen bonds with both the ribose and a nucleobase. These interactions seem to be conserved in all dsRBDs. We noted that instead of M371 in the human dsRBD at dsRNA recognition region 1, in some dsRBDs like those of *S. rosetta* (E426) and *F. alba* (Q410), the hydrophobic residue is replaced by a hydrophilic residue capable of interacting with the dsRNA, providing an additional anchoring point to the dsRNA. Although the majority of dsRNA recognition is ensured by interactions involving the dsRBD canonical fold, we showed that in hDus2, two positively charged arginines (R360/R361) act synergistically to recognize the tRNA. These two positively charged residues are also present in *S. arctica* (R426/K427) and *S. rosetta*. In contrast, only one of these positively charged residues is observed in *F. alba* (R400) and *A. queenslandica* (K381). Collectively, all dsRBDs of Dus2 analyzed so far likely carry a dsRNA binding capacity via the cooperative action of both their canonical structure and NTE.

#### 3.4.3. Pyridoxamine 5′-Phosphate Oxidase Domain

This domain is typically that of an FMN flavoprotein that catalyzes the oxidation of pyridoxamine-5-P (PMP) and pyridoxine-5-P (PNP) to pyridoxal-5-P (PLP). This type of protein is involved in the last step of PLP cofactor anabolism. The structural alignment of the PyrOx domain of Dus2 from *P. pensylvanicum* with 1G79, the *E. coli* PyrOx shows a good conservation of the global folding (RMSD: 3.87 Å on 120 Cα), except for some loops whose length is more important in Dus2; however, the core, i.e., the central beta-sheet as well as the helices, are perfectly aligned (Figure 8C). The cleft that acts as an active site in PyrOx containing FMN and PNP is also present in the PyrOx domain of Dus2, however a difference distinguishes them. Although this cleft is large enough to accommodate both FMN and PNP in the PyrOx of Dus2, its hydrophobic nature does not allow the binding of FMN and PNP in Dus2 (Figure 8D). Indeed, the active site of the PyrOx is generally positively charged, which allows the stabilization of the phosphate groups present on the FMN coenzyme and PNP substrate. This polarity inversion raises serious questions about the exact role of this domain in Dus2. A more detailed analysis of this domain in Dus2 shows the existence of another large pocket above the hydrophobic crevice that is positively charged (Figure 8D). It remains to be seen whether this could serve as an RNA binding site, which would imply that the function of this domain has evolved to allow for RNA recognition, which does not seem incongruous given that the phosphates play a key role in enzyme/substrate recognition in RNA modifying enzymes. Although this remains speculative, it is an interesting hypothesis to test experimentally.

#### 3.4.4. A Newly Identified Rossman Fold Domain

We have been able to identify for the first time that all the Dus2 of Schizosaccharomyces species, which are four in number (*S. pombe*, *S. cryophilus*, *S. octoporus,* and *S. japonicus*), carry after their HD an additional domain of about 145 amino acids (Figure 3 and Figure 4). This domain is organized around a central β-sheet made up of four parallel β-strands surrounded by six alpha-helices. A quick analysis by Dali suggests that this domain adopts a Rossman fold type domain. Its functional role in RNA binding is unlikely since the analysis of the electrostatic surface does not delineate positive patches that are expected to be observed for RNA binding sites. At this stage, it is not possible to discard this possibility or a potential other role, such as its implication in a regulatory process, but this would require biochemical validation.

#### 3.4.5. ICL_KPHMT and CTNS Domains

Members of the ICL/PEPM_KPHMT enzyme superfamily catalyze the formation and cleavage of either P-C or C-C bonds. Typical members are phosphoenolpyruvate mutase (PEPM), phosphonopyruvate hydrolase (PPH), carboxyPEP mutase (CPEP mutase), oxaloacetate hydrolase (OAH), isocitrate lyase (ICL), 2-methylisocitrate lyase (MICL), and ketopantoate hydroxymethyltransferase (KPHMT). In Dus2, this domain adopts a TIM-barrel, with 8 alpha helices surrounding the central barrel (Figure 8E). This domain is connected to the *P. insidiosum* ZnFD by a long linker of 34 amino acids (S401-I435). The terminal beta-sheet of the barrel gives way to a helix of about 20 amino acids that is followed by three other small helices, thus completing the sequence of the protein. On the basis of the electrostatic surface, it is difficult to predict any role for this domain (Figure 8F).

In *Trichinella nelsoni*, the dsRBD of Dus2 is followed by a CTNS domain, a cystine/H+ symporter known as a mediator in the export of cystine, the oxidized dimer of cysteine, from lysosomes. Importantly, no structure for such a domain has been reported in the PDB database. In the case of *T. nelsoni*, the CTNS is connected to the dsRBD by a 27 amino acid linker (N475-I501) (Figure 5G). Interestingly, the CTNS domain of Dus2 has a highly hydrophobic central ring with a width of more than 25 Å that serves as an anchoring zone across the membrane (Figure 8G). This means that Dus2 in these organisms is localized to the cell membrane or to the membrane of a cell organelle. This is, to date, the first case of an RNA-modifying enzyme that has a transmembrane domain. The canonical domains of these Dus2 as well as the dsRBD are probably cytosolic to allow modification of RNAs. However, the precise localization of these Dus2 remains to be clarified. In humans, hDus2 has been localized in the endoplasmic reticulum [13].

### 3.5. The functional Role of Dus2 Extensions and the Evolution of tRNA Binding

**Figure 9:**
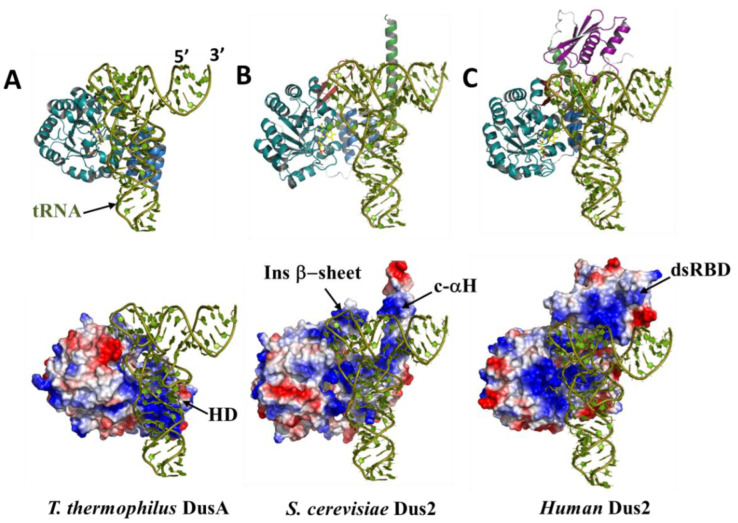
Evolution of tRNA binding mode in Dus enzymes catalyzing D20 biosynthesis. (**A**) Crystal structure of *T. thermophilus* DusA in complex with tRNA (PDB: 3B0V). (**B**,**C**) Structural models of *S. cerevisiae* Dus2/tRNA and hDus2/tRNA complexes, respectively. The TIM-barrel domain (TBD) appears in teal, while the helical domain (HD) is in blue, the inserted beta-sheet in red, the connecting c-αH in green, and the dsRBD in purple. The FMN coenzyme is denoted in yellow. The electrostatic surface of each Dus protein is shown below the protein or tRNA.

To determine whether these extensions have functional relevance in D20 biosynthesis, we conducted a comparative structural study of Dus/tRNA complexes from three different organisms (Figure 9 and Figure S9). The first one is *T. thermophilus* DusA, for which an X-ray crystallographic structure of a DusA/tRNA^Phe^ complex is available [22]. In this structure, DusA captures the tRNA in a positively charged crevice, where the HD contributes by providing most of the positive residues while others come from the catalytic domain (Figure 9A and Figure S9A,B).

Interestingly, no major conformational changes are observed between the free and bound states of either the protein or the tRNA substrate. Nonetheless, slight distortions of U16, U17, and U20 are observed, as is a flipping of the latter base, which found itself buried in the active site pocket stacked on the si-face of the isoalloxazine. DusA recognizes almost the complete tRNA elbow region formed by the junction of the D- and T-loops, stabilized by critical tertiary interactions, and the enzyme flips the target base without unwinding this tRNA structure. The absence of a drastic conformational change during catalysis in this family of enzymes is also supported by two DusC/tRNA structures [27]. These observations indicate that flipping of the target uridine to enter the active site can occur without altering the tertiary structure of tRNA, and this could be the case for the different types of tRNA substrates. Instead, these enzymes, as is the case for many tRNA-modifying enzymes, make use of a complementary surface to charge and shape the tRNA target region. In light of this information, we set out to model a *S. severvisiae* Dus2/tRNA complex. *T. thermophilus* tRNA^Phe^ from the DusA/tRNA structure was chosen because it is already in a productive conformation and ready for modification.

In order to generate this model, we aligned the DusA/tRNA^Phe^ structure with the *S. cerevisiae* Dus2 model. Remarkably, this alignment did not generate any major clashes; however, some slight manual readjustments were necessary. At first glance, *S. cerevisiae* Dus2 does not need to resort to some drastic conformational changes in its polypeptide to accommodate the tRNA (Figure 9B and Figure S9C,D). The TIM-barrel in cooperation with the HD, which carries several positive patches in a large crevice formed by the junction of the two domains, provides the tRNA binding site. Again, Dus2 embraces much of the elbow, the D- and T-loops. Surprisingly, the c-αH, which carries positive charges for more than half of its length, appears to be perfectly positioned to recognize an additional tRNA region (Figure 9B and Figure S9C,D), namely the back of the acceptor stem, whereas in the DusA/tRNA^Phe^ complex, this region is entirely free and solvent accessible. Thus, in cooperation with the canonical domains, this extension acts as a new tRNA anchor, allowing a broader recognition surface than that engaged by DusA. Finally, we examined Dus2 with dsRBDs, taking the human enzyme as our preferred choice because we have previous experimental data that allowed us to delineate the exact areas of interactions on both the protein and tRNA [29]. By reproducing the same approach used for *S. cerevisiae* Dus2, we were able to generate a structural model of full-length hDus2 in complex with tRNA (Figure 9C and Figure S9E,F). As for *S. cerevisiae*, a slight repositioning of the tRNA was required to remove the few collisions observed between the protein and tRNA. Interestingly, the resulting model is in close agreement with the previously proposed model using isolated domains of hDus2, which was inferred from mutagenesis, crystallography, NMR, and SAXS experiments [29]. The dsRBD recognizes a larger surface area of the tRNA acceptor region. Through the acquisition of this additional domain, hDus2 recognizes almost the entire tRNA molecule, except the anticodon region. Interestingly, c-αH no longer carries a positive surface charge since it is not involved in tRNA binding (Figure S9F). In a way, it is the dsRBD that compensates for this loss of charge compared to the yeast Dus2. Finally, it is worth mentioning that we have not been able to produce reliable models of the Dus2/tRNA complex with ZnFD-containing enzymes because this domain is not positioned in an orientation that prevents it from colliding with tRNA. However, it can be assumed that, as in all Dus, the canonical domains of these Dus2 should recognize the same tRNA regions as their counterparts. Under these conditions, ZnFD could, as with c-αH and dsRBD, recognize the acceptor stem and the T arm, both of which form the longest dsRNA region of the tRNA.

## 4. Conclusions

Artificial intelligence via AlphaFold has revolutionized structural biology due to the accuracy of the structural models generated by this methodology [50,51]. Combining this approach with phylogenetic analysis has proven to be an interesting strategy to study the evolutionary and functional features of enzyme systems. The application of this methodology to Dus2 from different organisms along the evolutionary tree and the analysis of the resulting structural models allowed us to unexpectedly discover that this enzyme presents a great deal of structural diversity through the presence of various extensions appended to the canonical fold that have an obvious functional relevance, at least for some of them. Analyses of enzyme/tRNA models perfectly illustrate the impact of the structural evolution of Dus on their tRNA recognition mode. What stands out from these models is that over the course of evolution, recognition of a larger surface area of tRNA by the Dus2 enzyme appears to have been deemed necessary for D20 biosynthesis. Although the analyses of protein surface electrostatics and modeling of Dus2/tRNA complexes suggest that some of these extensions are likely involved in RNA recognition, one can wonder the reason for such extensions from the standpoint of evolution. Recent publications have shown that eukaryotic Dus from *S. cerevisiae*, *S. pombe,* and humans can dihydrouridylate other substrates than tRNA, such as mRNAs, and long non-coding RNAs [4,5,6]. In bacteria, there seems to be no D in mRNAs and therefore the bacterial Dus may probably have only tRNA as a substrate. Thus, the need for D in other types of RNAs and consequently the additional substrates recognized by Dus2 could partly explain the increased complexity of the protein’s modularity compared to its bacterial counterpart. Another possibility could be an evolutionary shift towards a gain in stability of the Dus/tRNA complex during evolution, but this remains to be demonstrated experimentally.

In general, it is rare for tRNA-modifying enzymes catalyzing modifications targeting areas other than the anticodon to use this recognition mode. In contrast, enzymes that modify the anticodon use a tRNA recognition mechanism that involves large interaction surfaces [61]. This mode of recognition is also shared by the amino-acyl tRNA synthetases, which recognize almost the entire tRNA molecule. Moreover, this class of enzyme has seen its modularity increase in complexity during evolution by the decoration of additional domains, some for regulatory purposes [62]. More generally, there are many other examples of enzymes that gain modularity during evolution, with human proteins remaining the pinnacle of modularity complexity. However, in the case of tRNA-modifying enzymes catalyzing modifications in the tRNA body, the evolution of the structural diversity of Dus2 remains a unique example to our knowledge.

## Figures and Tables

**Table 1:** X-ray structures of Dus available in the protein data bank.

Proteins	Products	Domain/Complex	PDB Code	Resolution
*T. thermophilus* DusA	D20/D20a	full length	3B0P	1.7
*T. thermophilus* DusA	D20/D20a	full length + RNA fragment	3B0U	1.94
*T. thermophilus* DusA	D20/D20a	full length + tRNA^Phe^	3B0V	3.51
*E. coli* DusB	D17	full length	6EI9	2.55
*E. coli* DusC	D16	full length	3W9Z	2.1
*E. coli* DusC	D16	full length	4BFA	1.65
*E. coli* DusC	D16	full length + tRNA^Trp^	4YCP	2.55
*E. coli* DusC	D16	full length + tRNA^Phe^	4YCO	2.1
*Homo sapiens* Dus2	D20	TIM Barrel + HD	4XP7	1.9
*Homo sapiens* Dus2	D20	TIM Barrel + HD	4WFS	2.68
*Homo sapiens* Dus2	D20	dsRBD	4WFT	1.7
*Homo sapiens* Dus2	D20	dsRBD + dsRNA	5OC6	3.2

**Table 2:** Data collection and refinement statistics of the dsRBD of *A. queenslandica* Dus2.

	dsRBDaq
PDB code	8B02
**Data collection**	
Wavelength (Å)	0.9801
Resolution range (Å)	42.37–1.68 (1.70–1.68)
Space group	P2_1_
Cell dimensions	
a, b, c (Å)	29.077, 56.895, 63.587
α, β, γ (°)	90.00, 93.05, 90.00
Multiplicity	2.9 (2.0)
Completeness (%)	97.9 (79.7)
Mean I/sigma(I)	7.8 (0.8)
Wilson B-factor (Å^2^)	24.07
R-meas	0.090 (1.245)
R-pim	0.051 (0.762)
CC1/2	0.997 (0.351)
**Refinement**	
Reflections used in refinement	23356 (1170)
R-work / R-free (%)	20.35/22.78 (32.80/36.10)
Number of non-hydrogen atoms	
macromolecules	1548
ligands	28
solvent	139
R.m.s. deviations	
Bond lengths (Å)	0.009
Bond angles (°)	1.02
Ramachandran plot (%)	
favored	98.96
allowed	1.04
outliers	0.00
Average B-factor (Å^2^)	
Overall	28.45
macromolecules	26.91
ligands	42.77
solvent	42.75

## Data Availability

Raw data collection and refinement for the X-ray structure of dsRBD (PDB: 3B02) are available in the PDB.

## Supplementary Materials

The following supporting information can be downloaded at: https://www.mdpi.com/article/10.3390/biom12121760/s1, Table S1: List of Dus Family Subgoups; Table S2: Dus2 Fusions and Sequence Disorders; Figure S1: Example of Dus subfamily confirmation for Table S1 sequences; Figure S2: Example of Dus2 subfamily confirmation for Table S2 sequences; Figure S3: 3D-model obtained by AlphaFold2 of the Dus2 enzyme from different eukaryotic organisms; Figure S4: Structural alignment of AlphaFold models of selected Dus2 with the crystal structure of hDus2; Figure S5: Active sites alignment; Figure S6: Motifs conserved across Dus2 family members; Figure S7: Role of conserved residues interacting with the FMN; Figure S8: Predicted IDDT score per position for *S. cerevisiae* Dus2 models; Figure S9: Evolution of tRNA binding mode in Dus enzymes catalyzing D20 biosynthesis.
