# Supplementary material for: Evolutionary Diversity of Dus2 Enzymes Reveals Novel Structural and Functional Features among Members of the RNA Dihydrouridine Synthases Family
Source: Biomolecules. 2022 Nov 26;12(12):1760. doi: 10.3390/biom12121760 (PMC9775027; doi:10.3390/biom12121760)
Supplement: Supplementary file 1 [file biomolecules-12-01760-s001.zip › supplementary figures.pdf]

Supplemental Materials for Evolutionary diversity of Dus2 enzymes reveals novel structural and functional features among members of RNA dihydrouridine synthases Family

Murielle Lombard[1], Colbie J Reed[2], Ludovic Pecqueur[1], Bruno Faivre[1], Sabrine Toubdji[1,4], Claudia Sudol[1,4], Damien Brégeon[4], Valérie de Crécy-Lagard[2,3], Djemel Hamdane*[1]

[1]Laboratoire de Chimie des Processus Biologiques, CNRS-UMR 8229, Collège De France, Université Pierre et Marie Curie, 11 place Marcelin Berthelot, 75231 Paris Cedex 05, France.

[2]Department of Microbiology and Cell Science, University of Florida, Gainesville, FL, 32611, USA.

[3]University of Florida, Genetics Institute, Gainesville, Florida, 32610, USA.

[4]IBPS, Biology of Aging and Adaptation, Sorbonne Université 7 quai Saint Bernard, 75252 Paris Cedex 05, France

*To whom correspondence should be addressed:

Djemel Hamdane, Laboratoire de Chimie des Processus Biologiques, CNRS-UMR 8229, Collège de France, 11 place Marcelin Berthelot, 75231 Paris Cedex 05, France, Tel : +33-(0)1-44271642, Email : djemel.hamdane@college-de-france.fr

Figure S1. Example of Dus subfamily confirmation for Table S1 sequences. To confirm subfamily membership, sequences (50-100 at a time) were aligned and used to generate a sequence tree with control sequences. Red asterisks denote Dus1-4 control sequences, green asterisks denote DusABC controls.

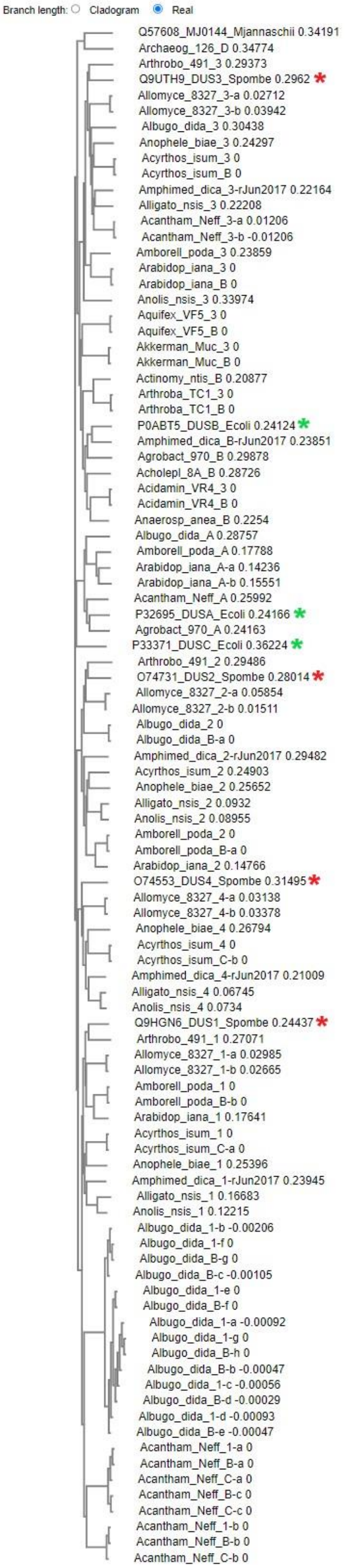

Figure S2. Example of Dus2 subfamily confirmation of Table S2 sequences. Each Dus2 sequence was double-checked for subfamily membership by alignment and generation of a sequence tree alongside 4 control sequences from *S. pombe* (Dus1-4). Red asterisks indicate tested/confirmed sequence.

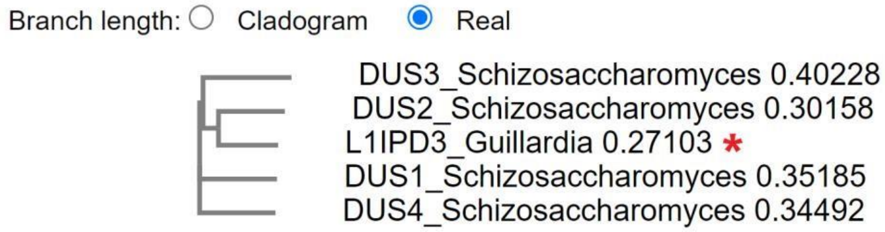

Figure S3. 3D-model obtained by AlphaFold2 of the Dus2 enzyme from different organisms

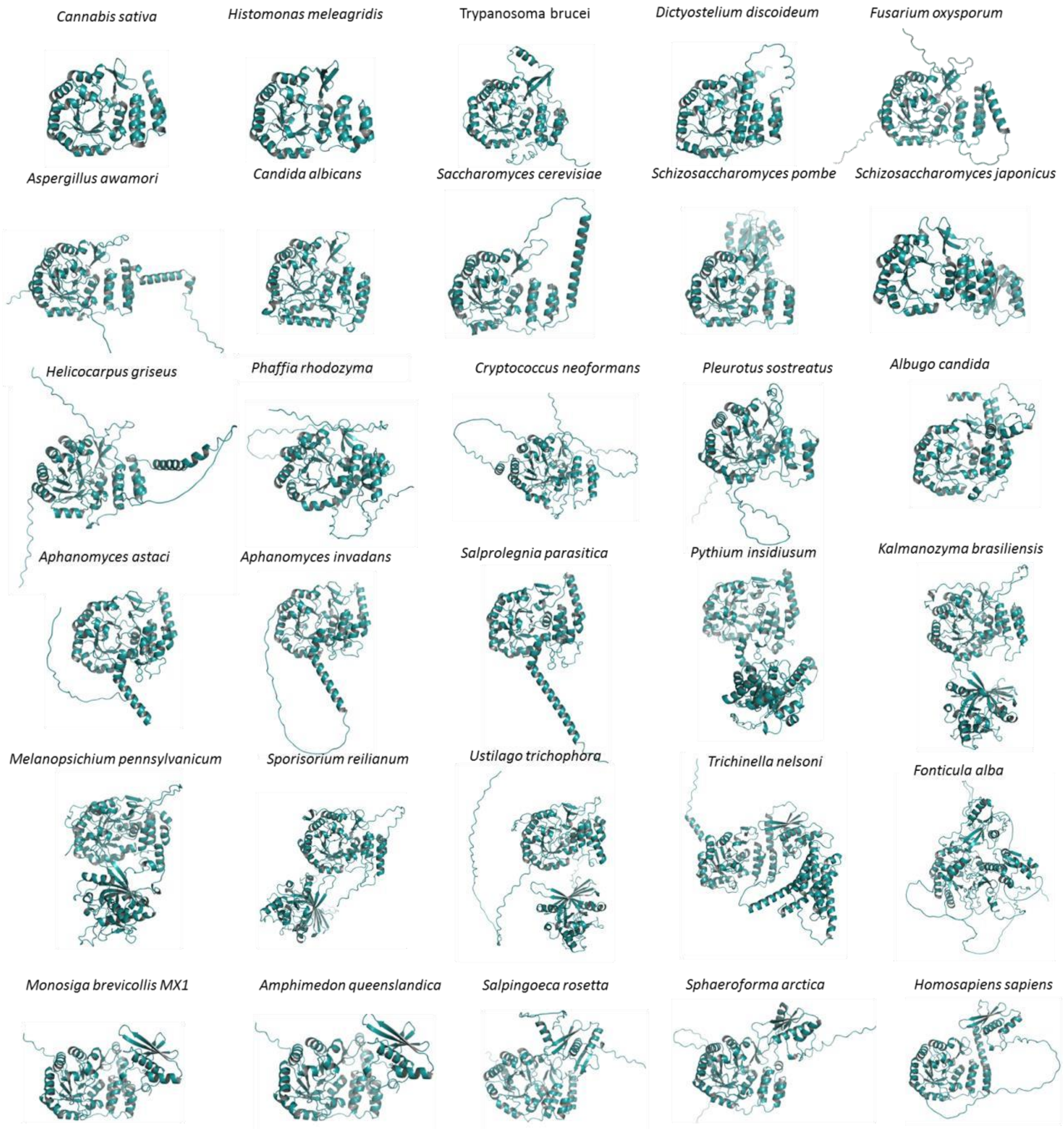

Figure S4. Structural alignment of AlphaFold model of selected Dus2 with the crystal structure of hDus2 (TIM-barrel + HD; PDB = 4XP7).

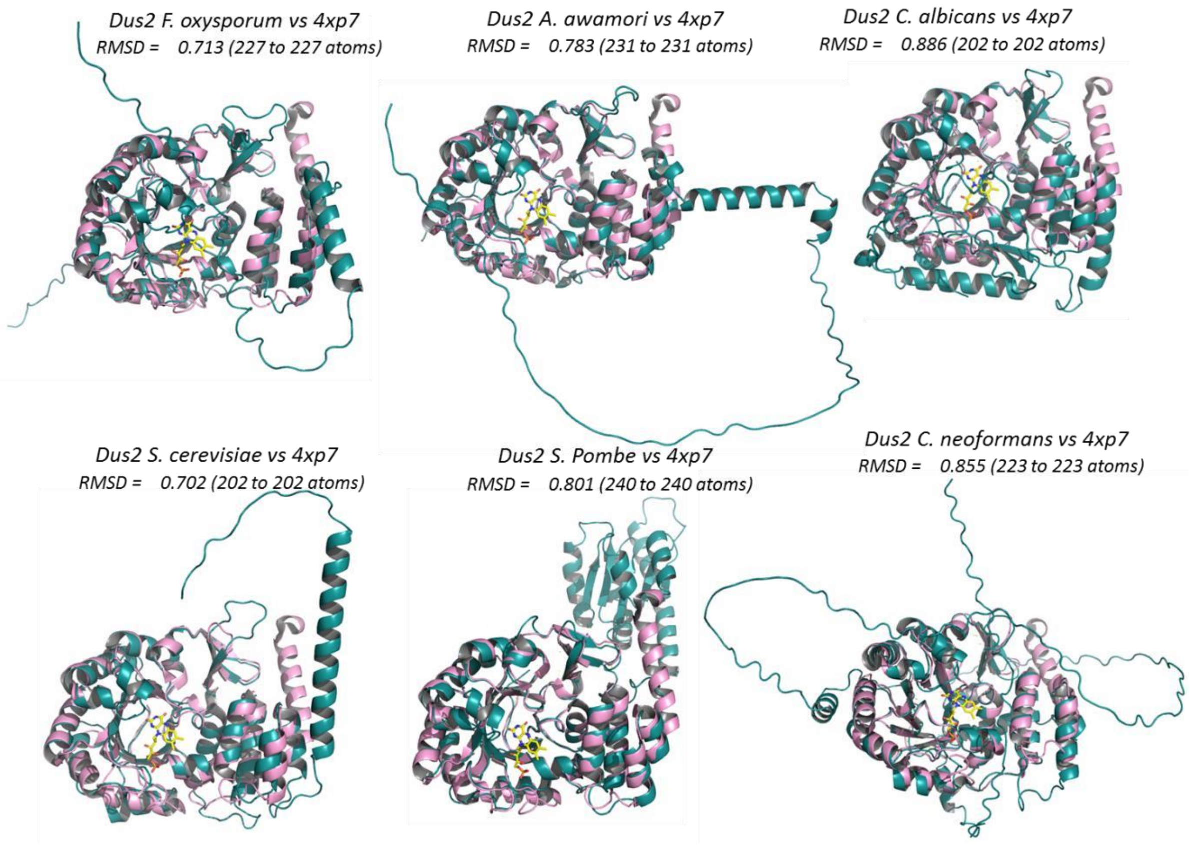

Figure S5. Structural alignment of active sites of selected AlphaFold model of Dus2 (colored in deepteal) with the crystal structure of hDus2 (PDB = 4XP7, colored in pink).

Dus2 ***S. cerevisiae*** vs 4xp7 RMSD = 0.702 (202 to 202 atoms)

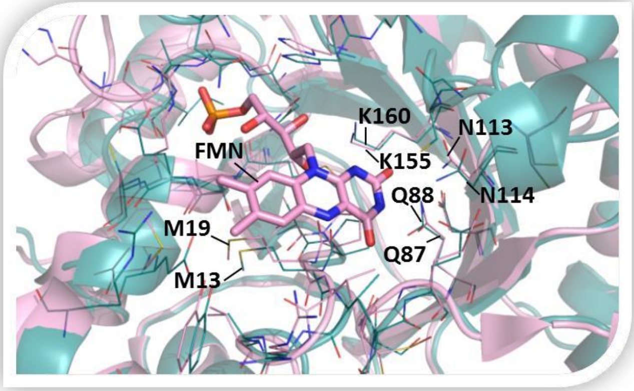

Dus2 ***C. albicans*** vs 4xp7 RMSD = 0.886 (202 to 202 atoms)

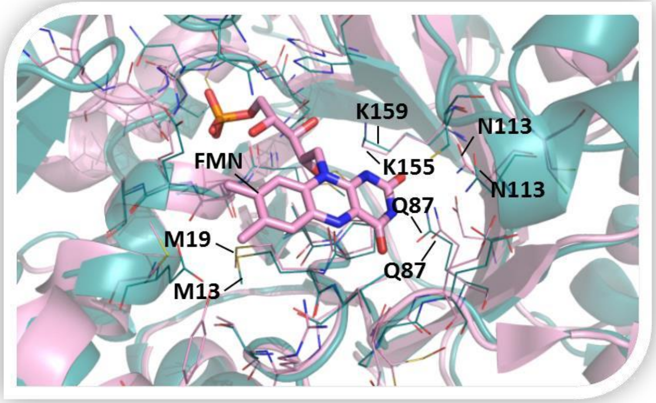

Dus2 ***C. pombe*** vs 4xp7 RMSD = 0.801 (240 to 240 atoms)

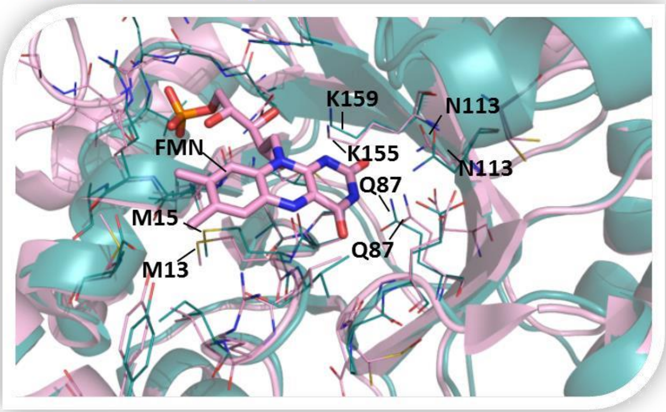

Figure S6. Motifs conserved across Dus2 family members. Active site residues are circled in yellow, key
residues are labeled with those highlighted in the structural analyses subsections.

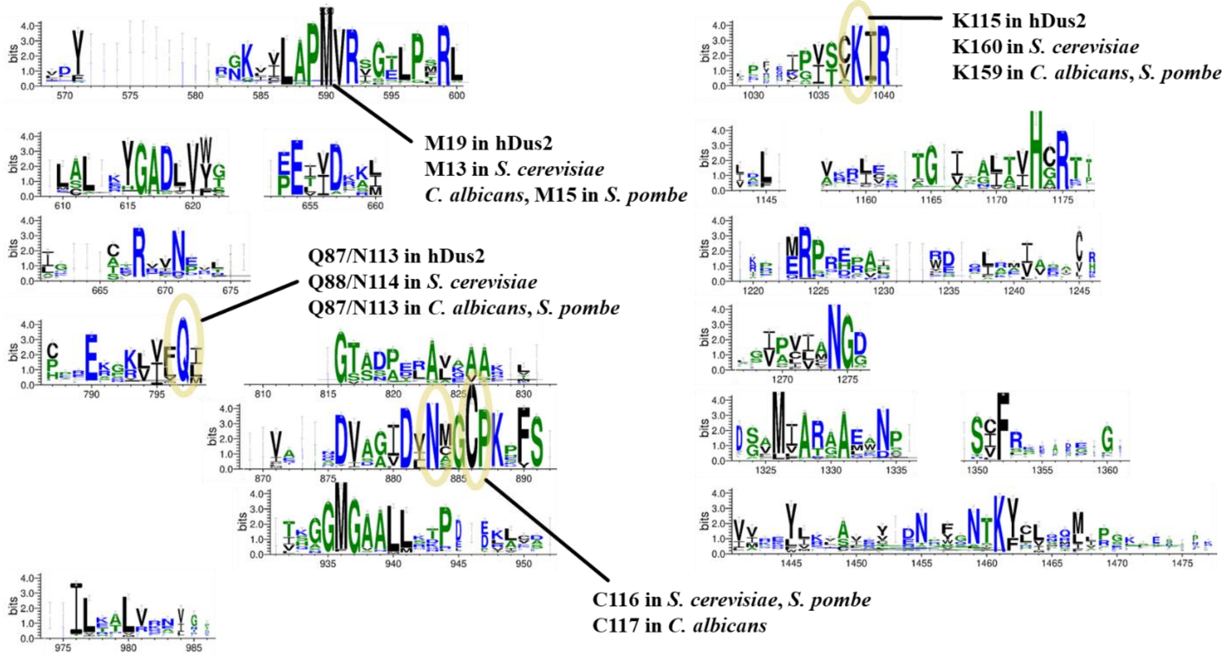

Figure S7. Role of conserved residues interacting with the FMN pyrimidine moiety in the stabilization of FMNH^{-}. The numbering of the residues is that of hDus2.

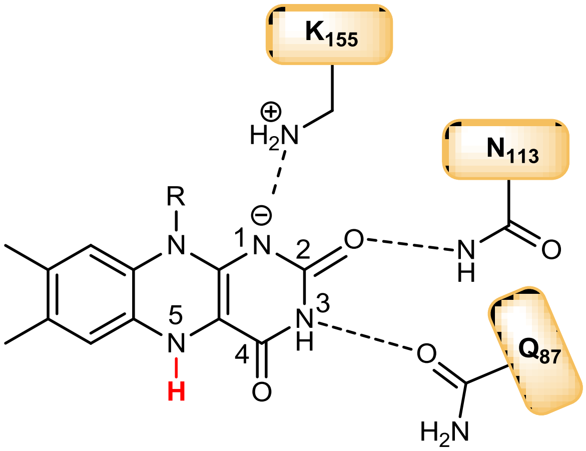

Figure S8. Predicted IDDT score per position for S. cerevisiae Dus2 models obtained from AlphaFold2.

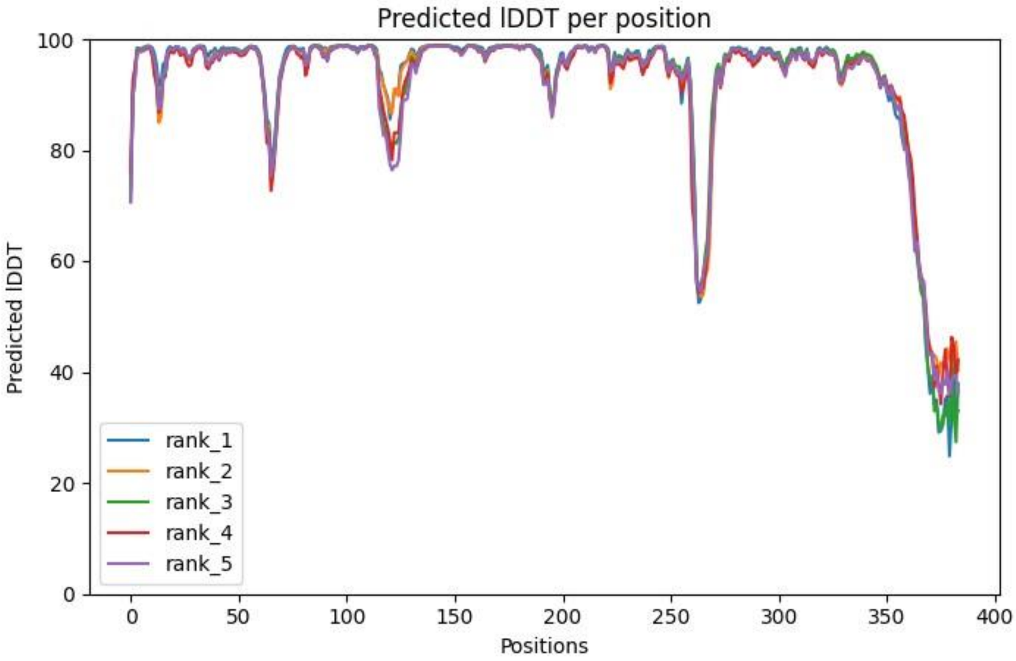

Figure S9. Evolution of tRNA binding mode in Dus enzymes catalyzing D20 biosynthesis. The electrostatic surface of each Dus protein is represented for each protein/tRNA complexes. (A) and (B) Crystal structure of *T. thermophilus* DusA in complex with tRNA (PDB: 3B0V). (C) and (D) Structural models of S. cerevisiae Dus2/tRNA (E) and (F) hDus2/tRNA complexes, respectively. The connecting c-αH and the dsRBD are framed by a dotted box. The tRNA cartoon is represented in green.

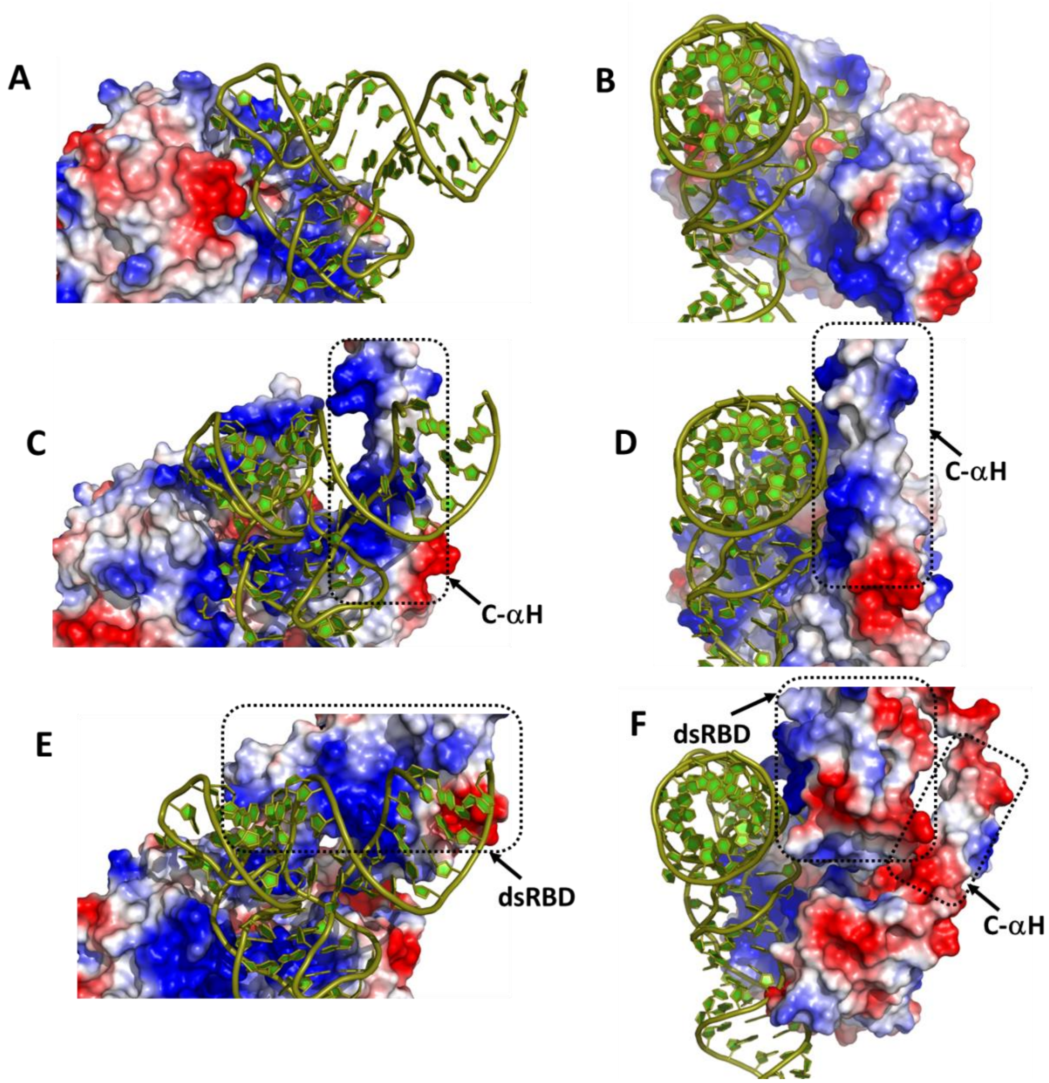

Supplementary Data Tables

Data Table S1. Dus Family Subgoups, Absence-Presence Derived from OrthoInspector Queries using Representative Sequences (see methods). Tax identifiers have been assigned based on their respective UniProt records. A column holding the specific organism's name is included and precedes the count data for each Dus subfamily, each of which possesses its own column.

Data Table S2. Dus2 Fusions and Sequence Disorder. Sequences curated from and merged between OrthoMCL, InterPro, and Pfam (see methods). Fusion "namesakes" assigned through CDD batch analysis (high specificity hit(s) of non-Dus domain(s) to identify respective Dus2 fusions). Similarly to the former dataset, taxonomic identifiers were assigned using UniProt records of respective accession entries. Sequence lengths were also retrieved from UniProt entries, as well as the N- and C-termini absence-presence and respective lengths. The presence of both Dus domain-circumfixing regions was converted to binary, both as individual termini (i.e., "N-terminus" and "C-terminus") and as a joint-presenting pair (i.e., "TwoRegions"). N- and C-termini were examined for disorder using InterPro. The absence-presence of disordered regions was also converted to binary (i.e., "Ndisorder", "Cdisorder).
